# Evaluation of a domestic violence training and support intervention in Palestinian primary care clinics in the west bank: a mixed method study

**DOI:** 10.1186/s12875-025-02751-y

**Published:** 2025-04-04

**Authors:** Nagham Joudeh, Amira Shaheen, Loraine J. Bacchus, Manuela Colombini, Abdulsalam Alkaiyat, Helen Lambert, Rasha Hashlamoon, Gene Feder

**Affiliations:** 1 Public Health Department, Faculty of Medicine and Health Sciences, An-Najah National University, P.O. Box 7, Nablus, West Bank Palestine; 2 Department of Global Health and Development, London School of Hygiene & Tropical Medicine, Faculty of Public Health & Policy, 15-17 Tavistock Place, London, WC1H 9SH UK; 3 Centre for Academic Primary Care, Population Health Sciences, Bristol Medical School, University of Bristol, Canynge Hall, 39 Whatley Road, Bristol, BS8 2PS UK

**Keywords:** DV, GBV, Primary care, Mixed method, Palestine

## Abstract

**Background:**

Domestic violence (DV) is a violation of human rights and a major public health problem that damages the health of women and their families. In the occupied Palestinian territories, 29% of women have a lifetime exposure to intimate partner violence, the most prevalent form of DV. Despite the existence of national policies to prevent and respond to DV, implementation within the Palestinian primary health care system has been weak. We developed, piloted, and evaluated a system-level intervention, including training for health care providers and care pathways for women patients. The aim of our evaluation was to determine the feasibility and acceptability of the HEalthcare Responding to violence and Abuse (HERA) intervention.

**Methods:**

Formative phase: adaptation of a previous (HERA) intervention implemented in primary health care settings in Palestine, informed by stakeholder meetings, interviews with clinic managers and health care providers (HCP), facility-level readiness data, and findings of a previous pilot study. The training component of the intervention, delivered by the Palestinian Counseling Centre, included a train-the-trainer session, two clinic-based training sessions, and reinforcement sessions for front-line healthcare providers in four clinics. Intervention: Healthcare providers were trained to ask about DV, give immediate support, and offer a referral to a nurse case manager. The care pathway beyond the case manager was either referral to a primary-care based psychologist or social worker or to a gender-based violence focal point external to the clinic that coordinated referrals to appropriate external services (e.g. police, safe house, psychologist, social worker). Evaluation phase: Thematic analysis of post-intervention semi-structured interviews with (HCP) and trainers; observations of training sessions and field notes. Provider Intervention Measure (PIM) data on changes in HCP attitudes and practice were analysed with descriptive statistics. Identification and referral rates for women disclosing DV 12 months before and 12 months after the intervention were obtained from clinic registries. We developed a theory of change to triangulate our qualitative and quantitative data.

**Results:**

The training proved acceptable to HCPs and there was evidence of positive change in attitudes and readiness to engage with women patients experiencing DV. Compared to the year before the intervention, there was a reduction in the number of patients disclosing DV during the intervention and of referrals in three of the four clinics. This reduction may be explained by the impact of the Covid 19 pandemic on clinic priorities, lack of time, persisting HCP fear about engaging with DV, and HCP rotation between clinics.

**Conclusion:**

The delivery of the training component of the HERA intervention within the Palestinian primary healthcare system proved partly feasible and was acceptable to HCPs, but contextual factors limited HCP implementation of the training in practice.

**Supplementary Information:**

The online version contains supplementary material available at 10.1186/s12875-025-02751-y.

## Background

Domestic violence (DV) - abuse perpetrated against an adult by an intimate partner or a family member - is a violation of human rights and a major public health problem that damages the health of women and their families. In the occupied Palestinian territories (oPT), 29% of women have a lifetime exposure to intimate partner violence, the most prevalent form of DV [1]. Only 5% of survivors seek formal help from the police or legal services despite a public awareness of support services [2]. In Palestinian society, gender discrimination remains common. Women are positioned as subordinate to men whose perceived value and power are represented in their greater access to material, symbolic and relational resources. In addition, the reality of Palestinian lives, including gender relations and gender dynamics, has been shaped by the prolonged Israeli occupation. There is a direct relationship between DV and exposure to occupation. Wives of Palestinian men directly experiencing political violence are at greater risk of DV than women whose husbands are not directly exposed [3]. Furthermore, women living in areas of the West Bank directly controlled by Israel (area C) and in refugee camps experience higher rates of DV and less access to health, social, and legal services because of restrictions on movement [4]. According to the Ministry of Health’s annual report for 2021, there are 765 of primary health care centres in the oPT: 606 in the West Bank and 159* in the Gaza Strip. 491 of these are run by the Ministry of Health (MoH), 65 by United Nations Relief and Works Agency (UNRWA), 192 by non-governmental organisations (NGOs), and 17 by the Palestinian Military Medical Services (PMMS).^1^

Eliminating all forms of violence against women and girls, which includes DV, by 2030 is integral to the UN Sustainable Development Goal on gender equality (SDG5), which would make a major contribution to improving the health of women. Integrating responses to DV within the health sector [5] has become a global priority [6]. In the oPT and globally, primary health care providers (HCP) have a unique place on the frontline of patient care in public, private and NGO sectors. This affords them the opportunity to engage with women survivors and offer them support. The oPT has a national referral system (NRS) for DV aiming to provide a comprehensive framework for coordinating referral to services for DV survivors across various public sectors including the MoH, Ministry of Social Development, the police, as well as NGOs [7]. In 2016, The MoH developed a policy framework for a primary health care response to DV, which was implemented in some clinics in the West Bank, including identification of cases and referral to external support services. However, the policy lacked comprehensive staff training and effective coordination for referrals [8]. Despite the existence of national policies to respond to DV, implementation within the primary health care system has been weak [9]. 

This paper presents a study that evaluated the feasibility and acceptability of HERA (HEalthcare Responding to violence and Abuse), a primary care-based intervention that included DV training for HCP and a referral pathway for women. The study aimed to identify the barriers and facilitators to implementation.

## Development of HERA intervention

The programme was designed to strengthen HCP capacity to respond effectively to women experiencing DV who present in primary healthcare settings. HERA adapted the IRIS (Identification and Referral to Improve Safety) intervention that was originally developed and evaluated in primary care settings in the United Kingdom [10, 11]. We piloted the adapted training programme in two MoH primary care clinics in the West Bank of the occupied Palestinian territories (oPT) in 2018 [12].

A key finding emerging from the pilot was the need to address structural factors within the primary care system, and especially to engage managerial support for the implementation of the intervention both for the training for health care providers and referral pathways. An additional finding was the need to increase clinician competence in providing psychosocial support for women who disclose DV, particularly those not wanting referral for other support. We undertook further intervention development to address these issues in parallel with linked studies in Nepal, Sri Lanka, and Brazil [13]. 

 World Health Organization [14] there was multilateral discussion between research collaborators in all countries and with the HERA global advisory group.

In order to obtain the support and input of key stakeholders in the oPT, we organised three meetings to discuss the structure and content of the intervention. The stakeholders included representatives inside and outside the primary health care system whose support was important for addressing contextual factors that could enable or obstruct the intervention. Stakeholders included the director of each clinic, the nursing director of each directorate, overall directors of the directorates, gender-based violence (GBV) focal point staff of MoH, representatives from the ministries of Health, Social Development, and Women’s affairs, the director of nursing in the MOH’s primary health care department, Tanmiya wa Aalam Almaraaتنمية وإعلام المرأة (TAM) Women Media and Development, the Palestinian medical council, the General Union of Palestinian Women, an expert on gender laws, an Al-Haq organization lawyer and the Palestinian Counseling Centre (PCC). Within the stakeholder meetings, the research team presented key findings from the formative phase interviews (described below) with HCP and managers regarding their views about the components of the intervention and the need for clear role definition in relation to DV response.

In recognition of the need for more psychosocial support for women experiencing violence and for expertise in delivering training to clinicians, we commissioned the Palestinian Counselling Centre (PCC) to co-develop the revised training content, particularly in relation to how to manage the psychological consequences of DV. The content was informed by the WHO *Health care for women subjected to intimate partner violence or sexual violence clinical handbook*, which includes a session on basic psychosocial support and identifying patients who need referral for specialised mental health care [15]. 

**Fig. 1:**
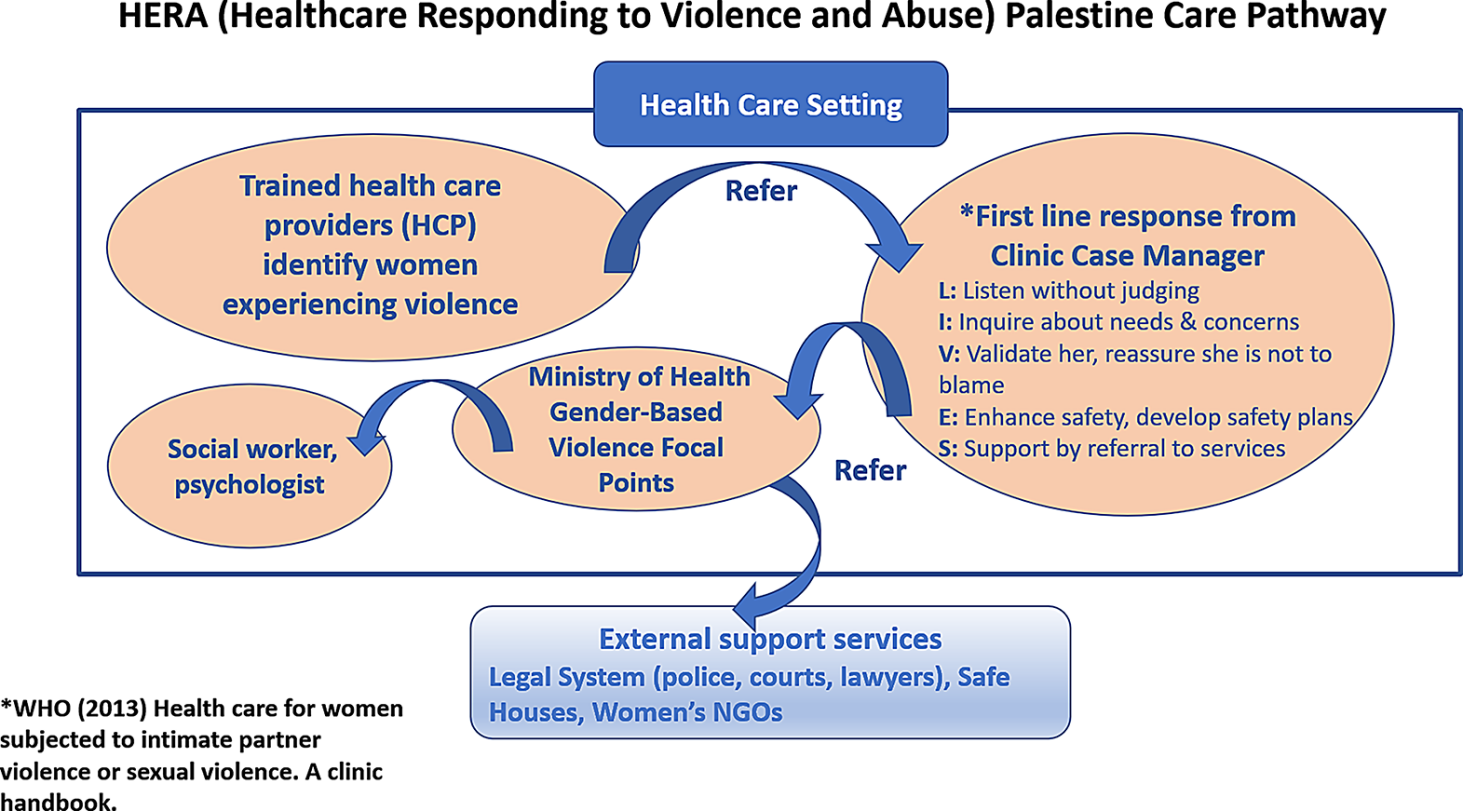
HERA Palestine care pathway

A new feature of the modified intervention was the inclusion of local professionals with DV expertise in HCP training: clinic case managers, social workers partly based in the pilot clinics and specialist gender based violence (GBV) nurses (‘focal points’). The focal points coordinate further internal (MoH) and external referrals for survivors of GBV. Figure 1 depicts the referral pathway and roles of different professionals.

Further features of the modified intervention were the introduction of mechanisms to address unavailability of HCP due to rotation between clinics and timing the training to be compatible with clinical responsibilities.

The final structure and content of the training emerged through dialogue between the research team and the PCC with input from staff in MoH GBV focal points. Training consisted of four train-the-trainer (case managers, GBV focal point staff and social workers) days, followed by two 2-hour HCP (doctor, nurse midwife, laboratory technician, clinic managers) training sessions, with monthly reinforcement sessions over six months.

Any HCP working in a primary reproductive health care setting, including maternity, family planning and vaccination services was eligible for the HERA intervention.

In our study we addressed the following research questions:

1. Did the intervention increase the confidence and readiness of HCP to inquire about, document, and offer a first-line response to women experiencing DV?
2. Did women have trust in HCPs and want to confide in them, and did they feel safe to seek help from services offered by the clinic and externally?
3. Did the intervention increase the identification of women experiencing DV and referral from HCPs internally to a case manager and from the case manager to external support services?
4. What factors challenged or supported the implementation of the intervention?

## Methods

### Settings

Our criteria for choice of the MoH clinic sites were (i) serving relatively socioeconomically deprived communities (ii) providing sexual and reproductive health (SRH) care services. Four primary care clinics were chosen in four different cities in the West Bank of the oPT.

Our original plan was to focus on sexual and reproductive health care providers, but we increased the scope to all primary health care providers, as there were insufficient numbers of the former and because other clinicians in these clinics provide sexual and reproductive health care. Maternal and child healthcare providers were also included, hence two pediatricians were trained. In the smallest clinic, all staff were included.

**Fig. 2:**
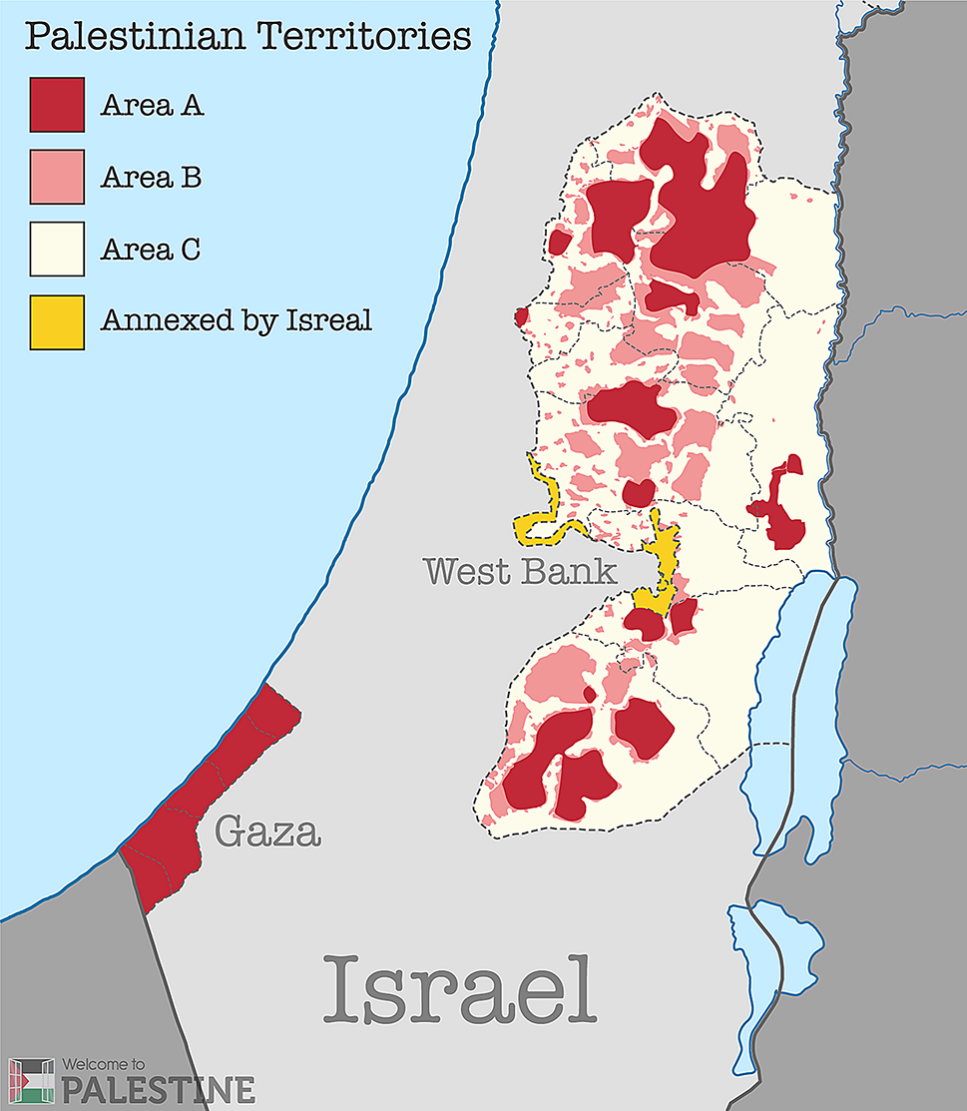
Palestinian territories; Areas A, B, C and areas annexed by Israel, Image reproduced with permission from Welcome to Palestine

The oPT has been split into three areas with different governance. Area A is administered by the Palestinian Authority, Area C by Israel, and Area B is under joint control. Illustrated in Fig. 2. The four clinics were located in areas A and C. We have chosen not to identify their location to protect the anonymity of participants.

### Study design and data collection

This parallel mixed method study [16] was conducted using the following quantitative and qualitative types of data collection:

### Provider intervention measure (PIM)

This survey tool (PIM) [17] measures the readiness and practices of HCPs in relation to identifying and responding to women experiencing DV. After forward translation of the tool into Arabic, cognitive interviews using a think-aloud method [18] were conducted with three health care professionals. No substantive changes were needed, although the feedback informed slight rephrasing of terminology and question structure. PIM was administered to clinicians attending the first training and readministered after the reinforcement session (6 months after the first training). See appendix for English and Arabic PIM.

### Qualitative interviews

After the training, semi-structured interviews were conducted with 20 HCPs and 2 trainers. HCP interviews were conducted at the clinics in a confidential environment, while the trainer interviews were conducted online. See appendix for topic guides.

Post-training interviews were conducted in the four clinics by NJ, RH, SSh, HKh, using the HERA topic guide in Arabic. Digital recordings were kept in encrypted files and transcribed by another research assistant. One transcript was translated to English.

### Field notes

RH, NJ, and AS kept field notes during visits to the intervention clinics including training sessions and informal conversations with HCPs, documenting attitudes and values as well as acceptability of the intervention.

### Identification and referral of women experiencing domestic violence

Clinic registries (log books) were used to collect data on identification and referral of patients with DV. Clinic data on DV cases were submitted to the national reporting system (NRS) which was in place before the start of the intervention.

The logbooks contained the following data about cases of DV: date of entry, serial number, file number, full name, type of visit (first time, recurrent), age group, marital status, perpetrator, recurrence of violence, type of violence, physical effects of violence, psychological effects of violence, medical procedures, referral, whether reported to the NRS, and notes of the HCP.

Before training, a designated HCP reported cases in the logbook provided by MoH. As part of the HERA intervention, this task was allocated to the case manager of each clinic. Doctors and nurses in each clinic were asked to report patients who disclosed DV to the case manager after patient consent. Extraction of identification and internal/external referral data from the logbook was performed for a one-year period both before and after the first training session. AS and NJ visited the clinics after the end of the follow-up period, copied the anonymised data entered into the logbook and uploaded the data into a password-protected file. Anonymised data were extracted from the copies of the logbook to an Excel spreadsheet.

### Analysis and results

The qualitative and quantitative data were analysed separately; integration of findings occurred at the interpretation stage [19]. 

Our data sources are summarised in Table 1 below. In the following section, we present analysis process and findings from the PIM questionnaire, starting with respondent characteristics, then presenting key findings and emerging themes from the qualitative interviews with health care providers and trainers. We then report identification and referral data, and conclude by integrating the different data sources to answer our research questions.

**Table 1:** Evaluation data sources: numbers of healthcare providers interviews, observed training sessions, pre and post PIM responses, trainers’ interviews

Data source	Pre-PIM	Post-PIM	post-training HCP interviews	Trainers interviews	Observed training sessions	Field notes
**Number**	23	22	20	2	25	-

## Quantitative analysis

We tabulated the response percentages for each PIM item for all pre- and post- intervention questionnaires. For the 10 HCPs for which we had matched pre- and post-PIM responses, we plotted individual differences between pre- and post-training in readiness, behaviors, and attitudes toward their role in responding to DV. Identification and referral data from the clinic logbooks before and after the intervention were summarised in aggregate and by clinic.

### Quantitative analysis results

Respondents to PIM and interview participants in all the clinics covered all the clinical roles and clinic intervention sides. (See Table 2)

**Table 2:** Demographics of pre- and post- intervention health care professionals PIM respondents and interviewees

**Pre-intervention health care professional PIM respondents**						
Pre-PIM						
Occupation	**General practitioner**	**Specialist doctor**	**Midwife**	**Nurse**	**Other**	**Total**
Number	3	3	4	8	5	23
Gender	**Female**	**Male**	**Unknown**			**Total**
Number	19	3	1			23
Age	**25–34**	**35–44**	**45–54**	**55-**	**Unknown**	Total
Number	4	6	9	3	1	23
**Post-intervention health care professional PIM respondents and interviewees**						
Post-PIM and interviewees						
Occupation	**General practitioner**	**Specialist doctor**	**Midwife**	**Nurse**	**Other**	**Total**
Number	1	2	3	7	9	22
Gender	**Female**	**Male**	**Unknown**			
Number	14	2	6			22
Age	**25–34**	**35–44**	**45–54**	**55-**	**Unknown**	
Number	1	9	3	4	5	22

### PIM

**Fig. 3:**
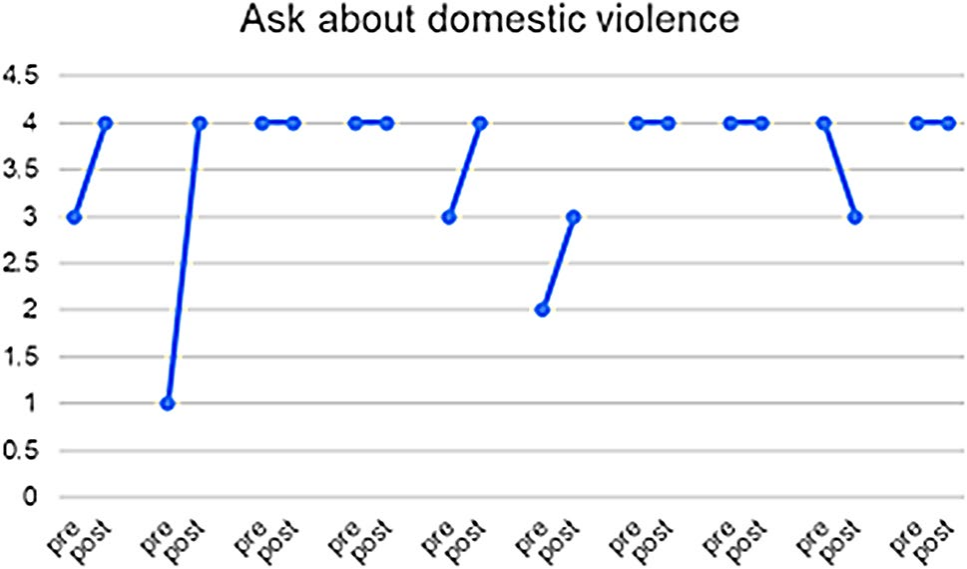
Q: How ready do you feel now (after attending the HERA training) to perform the following tasks when dealing with female patients who are experiencing domestic violence? ‘0’ indicates feeling “Not ready at all” and 4 indicates feeling “Completely ready”

**Fig. 4:**
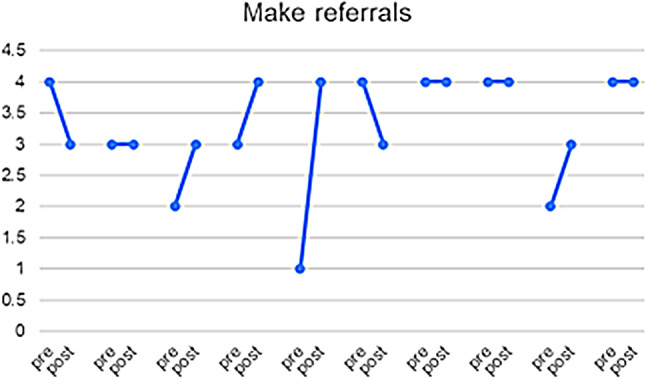
Q: How ready do you feel now (after attending the HERA training) to perform the following tasks when dealing with female patients who are experiencing domestic violence? ‘0’ indicates feeling “Not ready at all” and 4 indicates feeling “Completely ready”

**Fig. 5:**
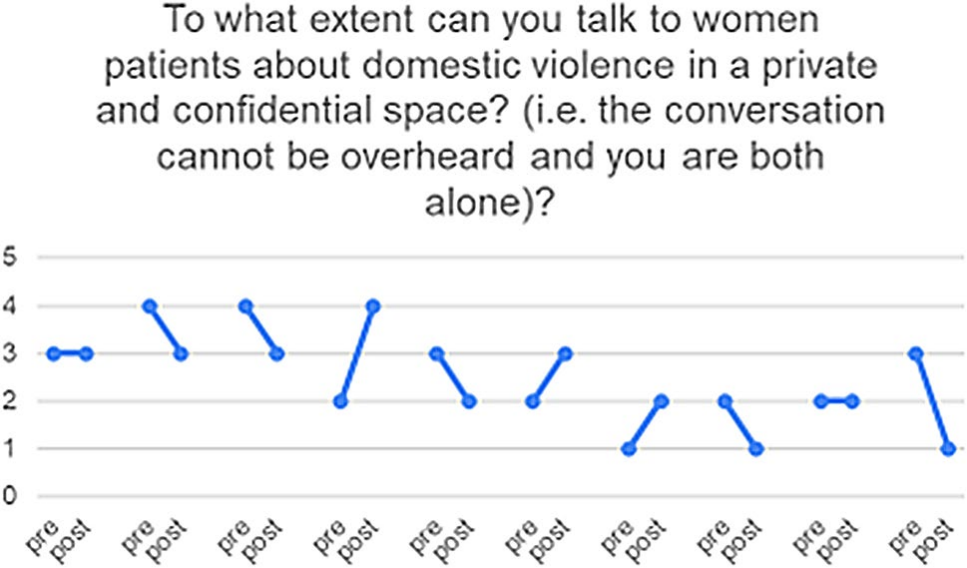
To what extent can you talk to women patients about domestic violence in a private and confidential space? (i.e. the conversation cannot be overheard and you are both alone)? (1) always possible (2) In most cases it is possible (3) rarely possible (4) never possible

**Fig. 6:**
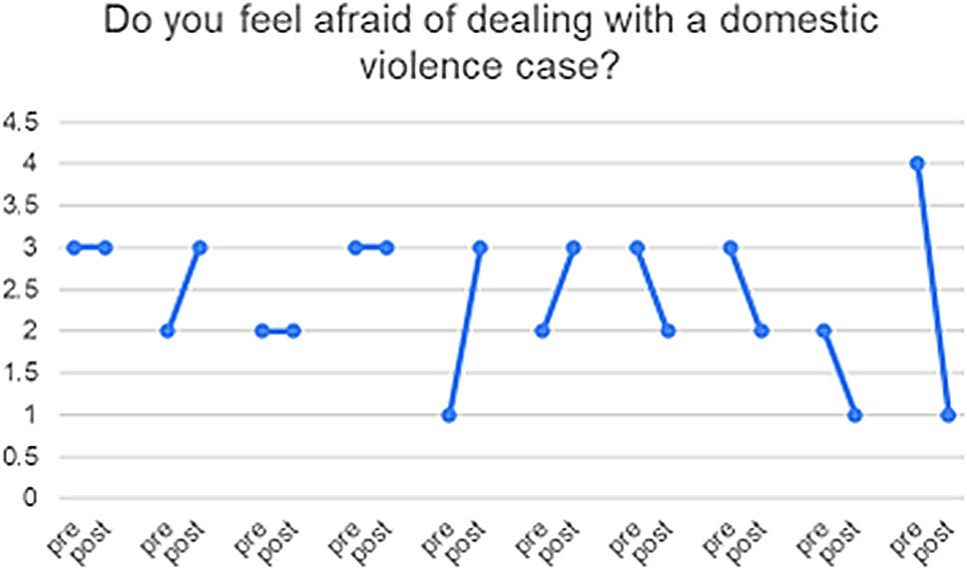
Do you feel afraid of dealing with a domestic violence case? (1) I feel very afraid (2) I feel moderately afraid (3) I do not feel afraid (4) I am not sure

**Fig. 7:**
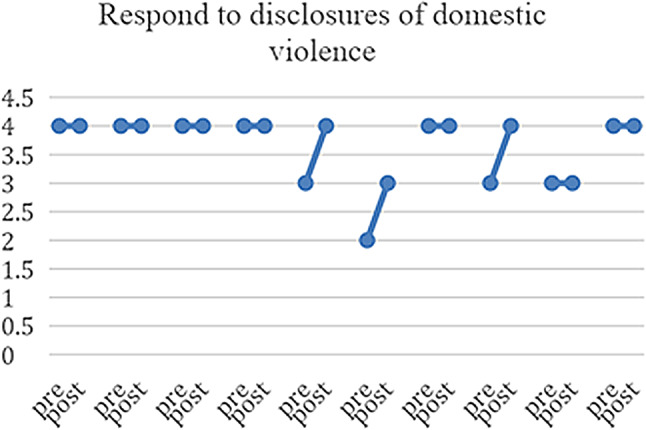
Readiness to respond to disclosure of domestic violence. ‘0’ indicates feeling “Not ready at all” and 4 indicates feeling “Completely ready”

10 of the 23 HCPs who responded completed both pre- and post-PIM questionnaires, enabling us to match the results for this subset of participants and explore the heterogeneity of the responses (see Figs. 3, 4, 5, 6 and 7) and (figures s1-s11 in the appendix). From these matched data, four HCPs reported being more ready to ask about DV, while only one reported being less ready, the remaining were unchanged. Four reported being more ready to make a referral when encountering a GBV case, while two reported being less ready. Three reported that they felt more afraid of dealing with DV cases while four reported the opposite. Three felt less protected by the clinic and four had no change. Five felt less able to talk to their patients about DV in a confidential environment.

Using data from all 23 respondents, there was an overall positive change in HCP reported readiness to identify and respond to women experiencing DV after the training (Table 2). HCPs reported being more ready to ask about DV, respond to disclosures, identify signs and symptoms, make referrals, and provide ongoing support to the women (see table s1 in appendix).

### Identification of DV

**Fig. 8:**
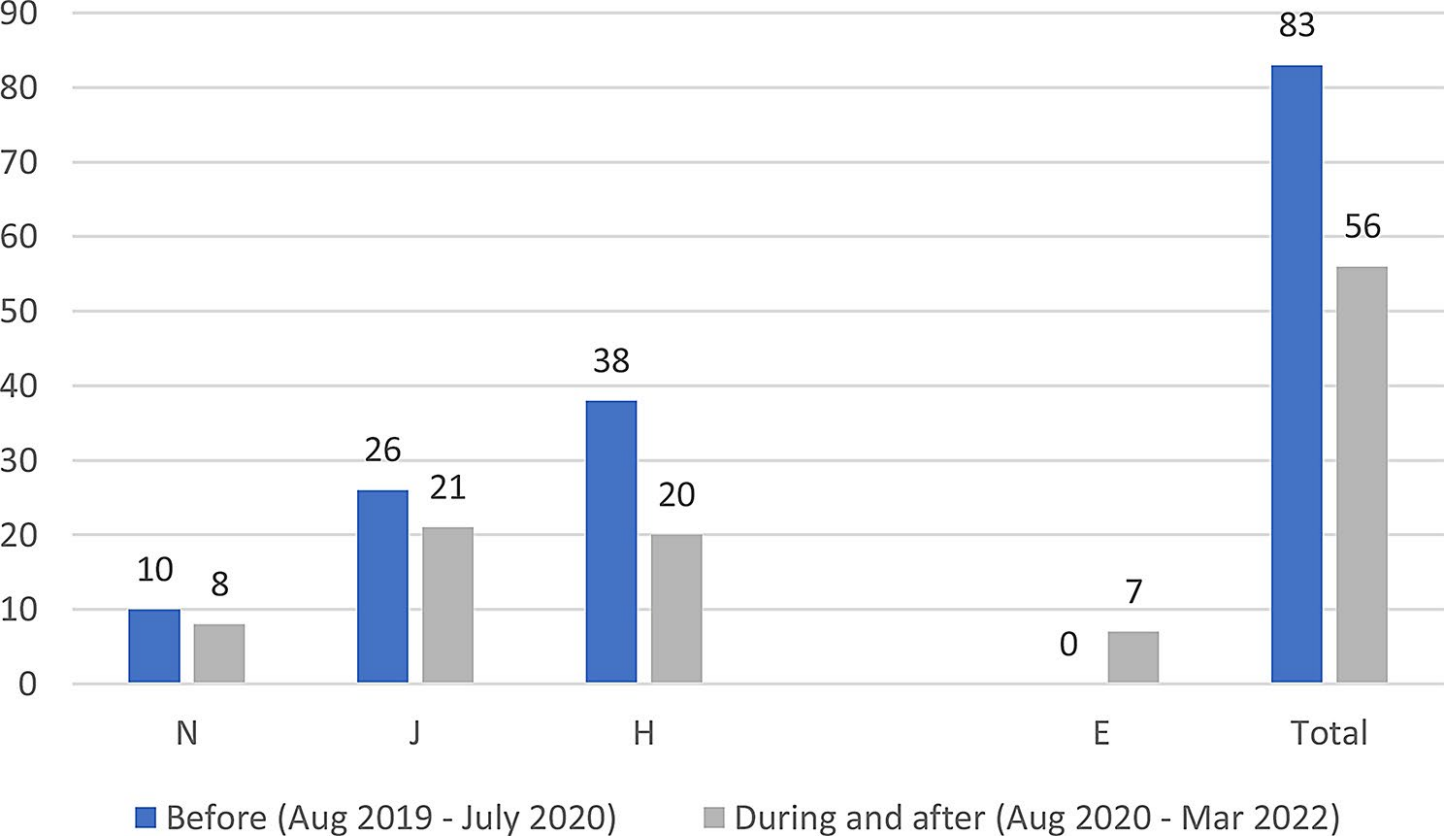
Number of DV cases identified in the participating healthcare settings before, during, and after the intervention

The logbook data showed that identification of women patients experiencing DV in the participating healthcare settings reduced in the year after the intervention started. Showed in Fig. 8.

### Referral

There were no referrals outside the clinics before the intervention and only one after the intervention.

### Qualitative analysis

Arabic transcripts were read and annotated by NJ and AS separately, then discussed and coded for themes which were grouped iteratively and then discussed in analysis meetings where codes were refined [20]. The interview translated into English enabled LJB to assist NJ in developing themes.

We used reflexive thematic analysis to identify patterns and themes in our data, following the approach outlined by Braun and Clarke [21]. This was chosen as it offered a flexible, yet systematic approach to thematic analysis that valued the researcher’s subjectivity as the primary way to discern meaning from the data. The interviews were transcribed verbatim, in Arabic, by a research assistant. The first step involved familiarization whereby the lead author (NJ) read and re-read all transcripts while annotating them with initial analytical observations and reflexive insights. Following this, the lead author systematically coded all interviews, generating codes that represented important features of the data. AS coded six interviews independently, while LJB coded one interview, in English, independently. The codes were examined by NJ and grouped to represent key themes and sub-categories within the data. Meetings between NJ, AS, LJB were conducted to validate emerging themes and their supporting quotes to ensure that they were grounded in the data, and answered the research questions. The themes were shared in an Excel Google document, reviewed and refined with input from the other co-authors, generating the main themes.

### Qualitative analysis results

The analysis generated five overarching themes, each with a set of sub-categories: (i) Contextual barriers; (ii) Impact on health care provider confidence and readiness to respond to DV; (iii) Implementation barriers; (iv) Supportive factors; (v) Sustainability.

### Contextual barriers

This theme is concerned with broader contextual barriers that affected the implementation of the HERA intervention: the Covid-19 pandemic, the ongoing Israeli military occupation, and cultural norms about gender roles and the importance of family.

### Covid 19

HCPs identified the Covid 19 pandemic as a major barrier to the delivery of the training and to implementing identification and referral of women patients experiencing DV.

HCPs talked about their experience during the lockdown. *“For a period of time*,* even the trainer couldn’t come to the clinic”* [nurse, female]. Their adapted schedule during the pandemic prevented them from attending the training as planned. *“Last year*,* there was a time when we only worked one day and off the other day (every other day)”* [nurse, female].

While HCPs believed that there was an increase in DV during the pandemic, the recorded number in the clinics either remained the same or decreased which they attributed to reduced clinic access.

*Our reports didn’t increase*,* unfortunately*,* they decreased*,* people were not able to reach [the clinic]. Afterwards there were many lockdowns and we provided one service- which is vaccination- and that continued throughout Covid. Other clinics were closed and only prescribed medications.… Here*,* the woman would just come to vaccinate her child in a hurry…* [GBV focal point, female].

This reduced access to the clinic may have also had a financial cause. *“Covid had an economic impact [negative]*,* affecting the survivor accessibility to clinics”* [specialist doctor, male].

Due to Covid restrictions, some training sessions had to be delivered virtually. However, HCPs reported a lack of managerial support to facilitate this transition.

*It was all on us*,* we would come each Saturday and Wednesday*,* there was this one time when we went into the central clinic and there were so many patients and no internet*,* and you have to make it work. So we got our phones and used 3G.* [GBV focal point, female]

Additionally, participation and engagement in the training sessions diminished when they were conducted online. One HCP emphasised how the lack of in-person interactions and non-verbal cues in online training negatively impacted attention and involvement.

*I prefer face-to-face training*,* because*,* as they say*,* the eye is the ladle of the soul. Body language attracts people (to be focused)*,* in contrast with the online session*,* you cannot focus on what’s being said* [nurse, female].

On the other hand, one trainer’s opinion on online training was that *“[I]t is clear*,* the same as face-to-face. There were some issues and resistance to start with*,* but at the end there was a general acceptance of the idea*,* it also gave a chance for more people to join*,* who couldn’t have joined otherwise.”* [Trainer2, male].

### Occupation-related safety and security issues

HCPs were concerned about the risk of violence from the families of women in areas where Palestinian police have no access. Security arrangements under the occupation prevent Palestine police protection in Area C.

*You know*,* in the small*,* faraway villages our personal safety is not guaranteed. The Israeli police are the ones responsible for the village area*,* and the Palestinian Authority isn’t able to come into it because they need permits* [nurse, female].

### Cultural norms

HCPs reported that DV is considered a private family issue, which sometimes affected the willingness of HCP to participate in the training.

*It is very difficult for a woman to open up with regards to a very sensitive issue such as GBV in a community that considers this issue as a family issue [2nd case manager/ Nurse*,* female].*

HCPs perceived traditional women’s role at home as a contributing factor to the difficulties in addressing DV. Their perception of DV as a private matter were sometimes evident in their expression of appropriate support.

*You know*,* when a woman has small children*,* our goal is not just to break up the family. Most just want us to listen and listen and give guidance. They want you to offer solutions. We tell them ‘That they must come up with the solutions themselves and we will help you get there.’ Once they tell us what they’re comfortable with*,* how they perceive their current situation*,* then we can provide more direction* [nurse, female].

### Impact on health care provider confidence and readiness to respond to DV

HCPs reported that the HERA training improved their documentation skills and gave some the ability to record DV work they previously did without reporting.

*Previously*,* we did not know that we should document everything we do with the cases. We do so much work*,* and we realized that it would be a pity not to document that*. *[2nd case manager/Nurse*,* female]*

It also made them more aware of their personal safety *“the training emphasized the fact that I should take my personal safety seriously*,* and not put my life in danger”.* [2nd case manager/Nurse, female]

HCPs reported improved confidence and readiness to inquire about, document and offer a first- line response to women experiencing DV. *“Before the training*,* we did not dare to deal with them (violence cases) or tell them to contact the police to ask for your rights. Now*,* we intervene more and we have more awareness and power.”* [Nurse, female].

The training also developed their perceived ability to build trust with women.

*Learning how to deal with and incidents from the very beginning*,* from the start*,* in reaching a certain point of trust between you [and the case]; we learned a lot. [2nd case manager/nurse*,* female]*

Finally, some reported improved skills in identifying and supporting patients experiencing DV.

*The subject is new to me*,* this is the first time I participate in something like this*,* I’m happy*,* given that I used to receive most of those cases*,* now I can identify them. Not all women will come to you and tell you I am battered*,* I am being hit*,* no one says it directly. [nurse*,* female]*

HCPs reported that they were confident about recognising patients experiencing DV, and picking up subtle signs like missing vaccination appointments.

*I notice sometimes from late (and not) coming to vaccination appointments. I follow and notice that. Vaccination is very essential*,* women here are rarely late for the appointment*,* only if their child gets sick*,* they postpone it for a week or two. But the ones who don’t show for a long time we call and ask about them*,* and tell them that we have a child that needs to be vaccinated*,* where is he/she*,* what’s up.* [nurse, female]

A wide range of physical signs took on a potentially different meaning. *“Either from the way she walks or how she cries*,* or when she sits on the patients’ bed we check her blood pressure and it’s high*,* or her weight is lower*,* she starts crying*,* but she doesn’t want anyone to know*,* that’s when we know that she is battered”* [nurse, female].

Other HCPs referred to information on patients’ financial situation as indicative of DV:

*I knew that one of my patients is experiencing violence when she wanted to open a family planning file. I asked her to get a receipt of 10 shekels*,* she stared at me*,* I asked her if there was something wrong*,* and I offered to give her the money if she didn’t have it. I explained that it wasn’t from me*,* and that there is a special box for that. Do you have the money? She answered: No*,* honestly my husband is so and so and he doesn’t give it to me.* [GBV focal point, female]

Overall, the trainers thought their training was well received, but were less positive about the external referral pathway.

*HCP fear to deal with GBV cases was reduced*,* not 100% though. Knowing the role of each*,* and emphasizing on the team work with the identified case increase HCP trust that they can provide help to survivors. Yet the issue of the trust was not eliminated*,* HCP lack trust in police and their managers to protect them from family retaliation.* [Trainer, Male]

### Implementation barriers

In the view of HCP and trainers, there were multi-layered barriers to implementing the HERA training and care pathway.

#### Patient-level barriers

HCsP reported that one of the barriers to disclosure was actually gaining trust, despite increased confidence to do it after the training *“Reality is always different from theory. It is not easy to get someone to trust you to tell you [about their case]. The practice was harder than the training that we received.”* [nurse, female].

HCPs reported that women have a fear of *scandal*.

*Once a woman came in*,* and her face was blue all over*,* she told me that it was in a road traffic accident. Me and the other nurse tried everything to get her to talk but she refused. She was scared of her husband and the scandal that will happen for her family and her children.* [midwife, female]

HCPs also reported that women hesitate to disclose abuse due to lack of privacy/confidentiality *“It’s not easy for a battered woman to disclose. Sometimes the nature of the place is not suitable*,* because it’s public you always have people coming and going.”* [nurse, female].

### HCP-level barriers

HCPs reported fear of retaliation and lack of safety.

*Interviewer: What could support you to make you feel safer? Respondent: Nothing. Interviewer: Not even security officers. Respondent: Will security come and protect me in my house? Of course not*,* someone might come and shoot at my house.* [midwife, female]

*There is not really personal safety for us in the clinic*,* unless the doctor clashes with anyone that tries to assault us. Him and the pharmacist are the only men we have around. It could be me at clinic*,* or me and [female nurse]*,* or just [female nurse] by herself with the doctor or pharmacist so*,* no there isn’t 100% personal safety for the medical staff.* [Nurse, female]

*Also there was a big resistance by a male doctor from another area who avoided GBV work and training completely because he worked in an area of clans.* [Trainer2, male]

This reference to clans implies that family disputes are mediated informally via families-based systems away from official and governmental involvement.

HCPs also reported that it is hard for them to give women time to disclose violence in a busy clinic setting.

*Some cases want more time and more effort as they are ready to ask for help and in some cases they don’t want to talk; you have to determine on your own if this woman is being exposed to abuse. That takes a lot of time.* [nurse, female]

Additionally, the discrepancy between what HCPs thinks a woman would benefit from versus what the woman wants for herself was mentioned:

*But one of our challenges is not reaching the result that we want. [Like that case] we wished we reached a farther stage with her*,* in helping her reach better circumstances*,* but the woman herself was unable to help us [get there].* [Case Manager, female]

In the trainers’ opinion, additional HCP barriers were *“lack of trust in their ability to help survivors*,* their beliefs about GBV*,* its existence and its nature”. [trainer*,* male]* They also mentioned initial opposition to training by HCPs. One trainer reported initial fears of HCPs as *“[H]elplessness*,* unclarity of roles*,* frustration for not being able to provide help.”* [Trainer2, male].

#### Organizational-level barriers

HCPs reported a lack of support from clinic managers that impeded their ability to participate in training and implement the intervention:

*Our managers don’t know anything about the programme*,* they didn’t participate in it or know any of the steps we’ve taken*,* or any details*,* they have not studied it. They only hear of it. They consider it trivial. When they allow you to go and attend a meeting [in GBV subject] they consider it secondary*,* among luxury. It is already hard work when you go to the training*,* but they make you feel like you’re going to enjoy yourself.* [GBV focal point, female]

One trainer also mentioned the low priority that clinic managers gave to the training, reflecting insufficient prioritisation of the response to DV.

*I was training the nurses to relieve their pressure*,* and it was hard because they were interrupted by their clinic work. Not only patients but also managers used to interrupt the HCP during the training*,* to ask them to do work in the clinic (working on clinic files).* [trainer, male]

HCPs also reported needing more time or dedicated space:*” When we see the cases*,* we need time to speak with them about their situation*,* our time is very limited and we have a high workload”* [Nurse, female].

#### Barriers to internal and external referral

HCPs reported women wanting to “talk” about their issues, but not access the internal referral to the clinic case manager: *“*They all say, don’t talk about it to anyone, we just want to talk (let it off our chest)” [nurse, female].

Also, some women were perceived as being fearful of external referral (to services outside the clinic). *“The issue is that you sometimes deal with VAW cases*,* but they don’t continue (with follow up)*,* they come for one or two sessions*,* and then she stops and gives in for her situation or when she feels that you are going to refer her to another institute they step back and don’t precede.”* [midwife, female].

Some also reported that the external referral destinations to be overwhelmed and women feeling *mentally exhausted* from the referral pathway and believing that the services outside the clinic are unreliable.

*You have to refer the woman to the social affairs*,* but they have so many cases. In the sequence*,* she must go to the family protection police. The woman feels that she is mentally exhausted*,* and that the service provided to her is not reliable and won’t solve her case 100%. Only when there is direct threat to her*,* family protection police intervene and then she is sent to a safe house. The governor/mayor or social affairs sometimes intervene to put her in safe place. But if a woman comes and generally mentions that her husband beats her a lot. I referred two to three cases to the social affairs*,* and they [social affairs] would say ‘what are we going to do for them*,* are we going to “nrabbi” [teach the right thing to do/ like parenting] her husband?’ [I replied] ‘Well*,* that’s your job*,* you have to go to her house*,* and check the situation.’ They go once and never go back. Afterwards*,* the woman would say that people [from the community/usually family] intervened and we solved it*,* she goes back to her initial situation. Nothing changed.* [GBV focal point, female]

#### Role-based differences in responses to training

Although clear role definitions for HCPs, case managers and GBV focal point staff were a key element of the intervention, this was only partly achieved in the training and further implementation within the clinics. For example, a trainer noticed that there was a poor relationship between GBV focal points and HCPs or social workers.

*The GBV focal point did not do her job in training the teams and explaining the roles. And that affected the team’s work. Nurses in the teams felt that they were not trained enough to start with*,* but as we went on in the training they showed more interest in discussion*,* and communication with battered women.* [Trainer 2, Male]

The trainer also mentioned that nurses were more interested in the training, while doctors had a more passive attitude. “Interviewer: Now I want to ask you about your experience with the training team, how do you assess the primary sessions and follow up? And how is training on site different than online? Interviewee: At first, the team used to say, what is this and why, Dr (1,female) was a a little bit angry and Dr (2, male) did not want to join from the start because he said it is not a part of his job and “authority”; the nursing team was more interested. We felt that the doctors “averted” and each time (training) they said they did not want to come. My female colleague (nurse) and I were more interested because this work was mostly on us.“ [midwife, female].

The objective of strengthening the relationship between case managers and GBV focal points was only partly achieved. This is evident from feedback provided by a GBV focal point, who highlighted that the training received by case managers was inadequate and that there was not enough time allocated for GBV focal points to provide additional training.

*But during this programme*,* my colleagues [in the Train the Trainers (TtT) supervision session] did not know much about the programme*,* and they did not have enough training to deal with battered women. They don’t know what violence against women is*,* as a basic definition*,* how are they going to do supervision in turn*… [GBV focal point, Female].

### Supportive factors

Interviewees reported several factors that were conducive to HCPs being able to participate in training and implement the intervention. One factor was role clarity; when this was achieved in the training this increased a feeling of safety in dealing with DV: *“I feel comfortable [about my safety in dealing with DV] because I know my boundaries really well.” [specialist doctor*,* male].* Similarly, the referral pathway was seen as successful because of clarity of roles: *“Referral pathway was successful because everyone knew their role.* [Specialist Doctor, Male]

Another supportive factor was formal evidence that the HCP had relevant expertise. One HCP reported that women have more trust in an HCP with credentials in GBV, related to the common assumption that women don’t think that GBV is part of the HCP role.

*“The certificate plays a role*,*…on what basis did you speak to the battered woman*,* I have a certificate and training experience*,* I have the right to talk to her*,* I’m not just anyone who came from the street who came to listen and talk to her*,* we have to have a certificate*,* something official to introduce ourselves with.”* [Nurse, Female].

### Sustainability

The absence of managerial support after the training was highlighted by a case manager as undermining sustainability of the HERA intervention. Whereas continued facilitation support from the PCC trainer was cited as a key to sustainability after the training:

*“HCPs are in continuous communication with the trainer either through the WhatsApp or the email consulting him regarding the identified cases. Two of the identified cases were referred to have treatment to the PCC where the trainer is working. In some cases*,* the trainer directs the HCP to the right place to where the case should be referred if it is not related to his specialty”* [midwife, female].

Long term sustainability would be dependent on stronger clinic and MoH managerial support and continuing engagement of the trainers in supervision. HCP cited the importance of ongoing facilitation and managerial support to sustain the programme: “*The important thing for us is to find someone who will continue working in this project.”* [Nurse, Female].

## Integration

**Fig. 9:**
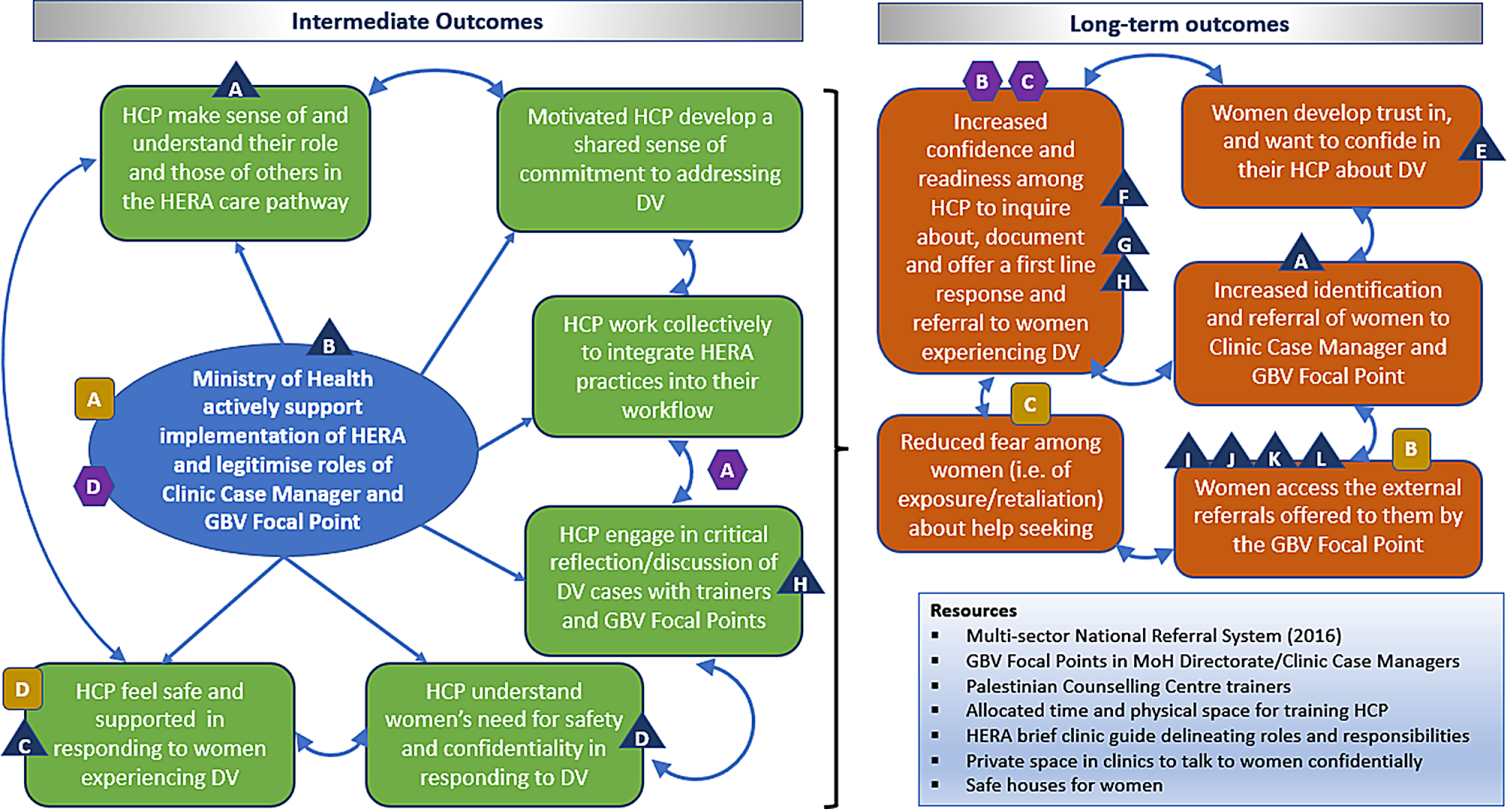
Theory of change for HERA intervention

**Fig. 10:**
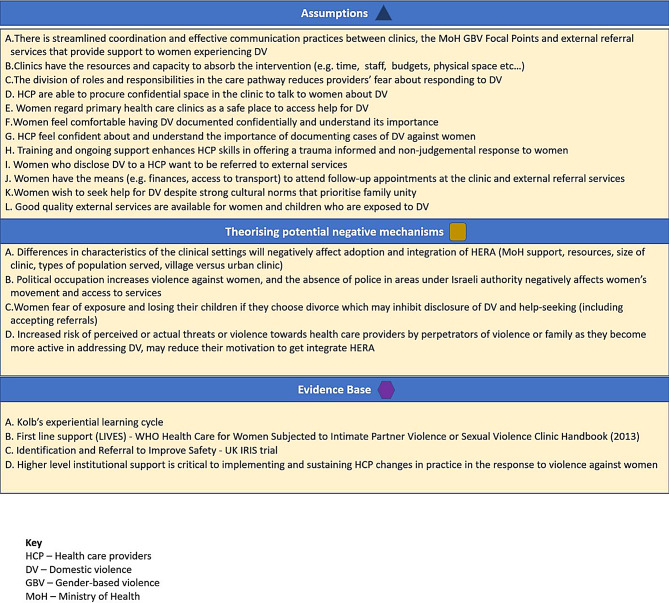
Assumptions and Theorising potential negative mechanisms of Theory of Change of HERA intervention

After the separate analyses of PIM, interview, and identification & referral data, we tabulated them by key assumptions and outcomes in the theory of change (ToC) that we developed in the course of intervention development (see Fig. 9 depicting the ToC which articulates how the intervention was expected to work at different stages (intermediate outcomes), and the conditions required to enable change (assumptions) (see Fig. 10), in order to achieve the desired longer-term outcomes [22].

For this analysis we also used observations from field notes. NJ and GF classified the evidence for the assumptions and outcomes as absent, present and inconsistent. This tabulation allowed us to judge feasibility and acceptability of the HERA intervention through the lens of the theory of change, specifically focusing on the barriers and facilitators of the intervention.

### Triangulation of qualitative and quantitative data

Table 3 represents the triangulation of the three data sources through the structure of the theory of change, making a judgement about evidence for its assumptions and outcomes.

**Table 3:** Triangulation of the three data sources (interviews, pre- and post-PIM, Identification and Referral data) based on assumptions and outcomes of the theory of change

Assumptions and outcomes from the theory of change	Interviews with providers & trainers and field notes	Pre- and post- PIM analysis/Identification and referral data	evidence (present, absent, inconsistent)
Increased confidence and readiness among HCPs to inquire about, document and offer a first line response to women and referral experiencing DV*	HCPs reported being more confident to deal with DV cases and they also reported that after the training they gained skills in building trust with women so they can disclose violence. They believed that their documentation had improved. HCPs reported that they felt more ready to make internal referrals after the training	There was increased confidence shown in matched pre- and post- PIM data in answers to: How ready do you feel now (after attending the HERA training) to perform the following tasks (ask about DV and make referrals) when dealing with female patients who are experiencing DV? (See Figs. 3 and 4)	present
Women develop trust in, and want to confide in their HCPs about DV*	HCPs reported that they were able to gain women’s trust and give them space to confide in their experience of violence	There was an overall decrease in identification of women experiencing DV (see Fig. 8)	Inconsistent
Increased identification & referral of women to case manager and the GBV focal point (MoH)*	Identification and referral numbers did **not** increase after the training. Mostly due to the Covid-19 pandemic, change in MoH clinic work days/priorities/responsibilities One doctor reported that they were increasing identification in their non-MoH clinical work. There was no increase in external referral. HCPs thought this was due to fear of external referral and women’s and HCP perception of unreliability of external referral destinations.	The number of cases identified and documented in clinics: pre-intervention: 83 during and post-intervention: 56 (see Fig. 8)	Absent
Women access the external referrals offered to them by the GBV focal point*	GBV focal point staff stated that few women experiencing DV accept external referrals	Only one external referral was made after training	Absent
Reduced fear among women (i.e. of exposure/retaliation) about help seeking*	HCPs reported that women who disclosed still feared retaliation from their husbands, family and causing a scandal		Absent
HCPs make sense of and understand their role and those of others in the HERA care pathway**	HCP reported that they better understood their roles and boundaries after the training		Present
Change in HCP values and attitudes about gender-based violence**	Several HCPs appeared to change their attitudes towards GBV and were more open to be a part of the health system response to it [from field notes]		Present
HCPs understand women’s need for safety and confidentiality in responding to DV**	HCPs reported becoming more aware of women’s need for safety and confidentiality	The decreased confidence by some HCPs about having a confidential space reflects an increased understanding of women’s needs of safety and confidentiality, rather than any change in the availability of those spaces. (See Fig. 5)	Present
Ministry of Health actively supports implementation of HERA and legitimises the role of clinic case managers and the GBV focal point **	HCPs reported that they did not have enough support to attend the training. (protected time, suitable place, logistics, etc.) MoH supported the creation of the new “case manager” role and assigned one for each of the 4 clinics but have not provided enough support		Absent
Motivated HCPs develop a shared sense of commitment to addressing DV**	Some motivated HCP reported that they felt a sense of duty to help women experiencing DV, but others did not.		inconsistent
HCPs work collectively to integrate HERA practices into their workflow**	HCPs followed the referral pathway in the clinics. According to trainers and HCP there was discussion of cases among staff, during the training and also later in practice		Present
HCPs engage in critical reflection/discussion of DV cases with trainers and GBV Focal Points**	During the training, HCPs engaged in discussions of DV cases encountered in the clinics with the trainers		Present
HCPs feel safe and supported in responding to women experiencing DV**	HCP reported fear of retaliation from DV victims’ families and perpetrators HCP reported that there is no protection or support from administration when they deal with DV cases Having official certificate/training legitimizes the HCP DV work and makes HCP feel safe	There were multiple trends of changes among matched pre and post PIM results answering the question: Do you feel afraid of dealing with a domestic violence case? (See Fig. 6)	Absent
There is streamlined coordination and effective communication practices between clinics, the MoH GBV focal points and external referral services that provide support to women experiencing DV***	HCPs, case managers and DV focal points reported effective coordination and communication practices along the referral pathway. However, external referral (outside MoH) was reported to be difficult and occasionally ineffective.		inconsistent
Clinics have the resources and capacity to absorb the intervention (e.g. time, staff, budgets, physical space etc…)***	HCP reported that there are limited resources for primary care clinics. There is a shortage of staff and increased workload. Additionally, some HCP reported not having the physical space for confidential disclosure of DV	Matched pre and post PIM results show decreased confidence by some HCP about having a confidential space (See Fig. 5)	Absent
HCP are able to procure confidential space in the clinic to talk to women about DV***	It was reported by HCP that in various settings there are no spaces for private and confidential disclosure of DV	Results for PIM question number 4 shows lack of confidential space (See table s6 in the appendix)	Absent
Women regard primary health care clinics as a safe place to access help for DV***	HCPs (+ case managers + GBV focal points) reported that women regard primary health care clinics as a safe place to disclose violence, and in some cases receive help	There is a documented reduction in identification (See Fig. 8)	Inconsistent
Women feel comfortable having DV documented confidentially and understand its importance***	HCPs reported that not all women are comfortable in documenting DV. Measures were taken to alleviate that including: securing confidentiality, using different names, keeping the clinic records in a safe place, restricted access to National registries of women experiencing DV.		inconsistent
Training and ongoing support enhances HCP skills in offering a trauma-informed and non-judgemental response to women***	HCP reported better skills in dealing and responding to GBV disclosure after the training	Matched pre- and post-PIM results show increased readiness to respond to disclosure of domestic violence (See Fig. 7)	Present

*long term outcomes

**intermediate outcomes

***assumptions

## Discussion

Our study has demonstrated the acceptability and feasibility, even during a global pandemic, of the training component of the HERA intervention in the oPT, but not of the referral pathway for further support. Our judgements about acceptability and feasibility are based on answering the four research questions that drove the study. First, the HERA intervention increased the confidence and readiness of HCP to inquire about, document and offer a first line response to women experiencing DVA, although not for all the HCP responding to the questionnaire or participating in interviews. Second, it is uncertain whether women patients trusted their HCPs with disclosure of DV and there was no evidence that they felt safe to seek GBV services offered by the clinic and externally. Third, identification and internal referral increased in one of the clinics, but decreased in the other three and referral to external support services did not occur. Fourth, a range of factors were barriers to implementation of the intervention, particularly the impact of the COVID-19 pandemic on primary care services. There was a marked variation between clinics, not just in numbers of patients experiencing DV who were identified by HCPs, but also in attitudes and effective communication between HCP and staff from GBV MoH focal points outside the clinic.

A qualitative systematic review exploring health professionals’ readiness to address gender-based violence suggests that when practitioners are motivated by a human or children rights perspective, a feminist lens or a personal experience of DV, their commitment to address gender‐based violence is enhanced [23]. This is consistent with the change we measured in readiness among HCP after HERA training and was clearly articulated in our interviews. DV training for HCPs may be effective for outcomes that are precursors to behavior change, such as attitudes towards responding to DV. It may improve DV knowledge and HCP self‐perceived readiness to respond to those affected by DV [24], although this evidence is uncertain. Although supportive evidence is weak and inconsistent, other studies have reported that training may improve HCP responses, including the use of safety planning, identification and documentation of DV in women’s case histories [25]. This is partly consistent with our findings that HCPs perceived improved readiness to deal with DV but gaps in readiness at HCP and organizational levels remained, particularly in relation to managerial support, a disconnect between what HCPs and women wanted, lack of privacy and resistance of women to referral for further support. This, was also a finding in a previous study of HERA implementation in the Palestinian primary care system [12].

HCPs had the following explanations for the lack of impact on disclosure, documentation and referrals: lack of accessibility (closed clinics during Covid 19 pandemic), women barriers (fear, cultural stigma, lack of trust in the system), HCP fears (lack of employer support, lack of safety measures, no police in certain areas due to Israeli occupation), organizational barriers (external referral destinations are unreliable and inefficient). Many of these barriers are ubiquitous across health services globally and exacerbated during the pandemic, but others, particularly the effect of the occupation, are specific to the oPT [26]. The individual, service, and broader contextual barriers to implementation of HERA articulated by HCPs were consistent with findings in a previous study where barriers to women survivors’ help-seeking for DV were reported [2].

The negative effect of the pandemic on case identification and referral was not mirrored by an impact on training, as this migrated online. The switch to online training was not easy for trainers or participants, but it proved effective. Trainers reported that it became a sustainable platform to train HCPs in areas they cannot reach physically. The detrimental effect of the pandemic on identification of patients experiencing DVA was multifactorial, consistent with our ToC that articulated contextual factors underpinning the HERA intervention. Repeated societal lockdowns not only closed clinics or re-directed their work; they also reduced freedom of movement for women, reduced resources needed to travel to clinics, and reduced confidence about travel and disclosure of abuse in clinical settings. Like other preventive health care, the response to DV was marginalised during the pandemic. In some countries, like the UK, these barriers were partly mitigated by online consultations with doctors [27]. This was not available in the Palestinian primary care system.

With reference to our ToC, although several of the assumptions and transitions were fulfilled, the overall decline in identification and the absence of referral shows crucial gaps that meant that the intervention could not improve safety and other outcomes for women experiencing DV. The HERA intervention in the oPT requires further development and testing for wider implementation. Key changes to improve implementation should include strengthening the integration of GBV focal points into HERA training and the care pathway, and protecting their time for that expanded role. Strengthening of the relationship between the HCP and the case manager is also necessary, with clearer role definition in relation to patients with DV at a managerial and policy level, increased valuing of the clinical response to DV, with equal priority with other services in the clinic. Recent MoH policy is clear about integrating DV into services, but this has not yet been operationalised. There is an urgent need to explore with MoH and police how to increase the safety of HCPs when engaging with patients, particularly in the context of DV and other stigmatised problems. Finally, regular DV training and reinforcement is essential to maintain competency.

Strengths and limitations.

Our mixed methods design enabled the triangulation of different data sources to test the acceptability and feasibility of the intervention. The ToC we developed allowed us to articulate mechanisms that provided a structure for the evaluation and interpretation of results. Our diverse sample of clinics was relatively representative of the population in terms of clinic type and location and of the range of HCPs. A notable strength was our persistence in conducting the study throughout the Covid 19 pandemic.

As with many public health interventions, the evaluation of HERA focused on practical application in the ‘real world’ rather than generating theoretical insights. The ToC provided valuable insights into the underlying mechanisms that helped or hindered the implementation process.

The study had several limitations including the absence of interviews with women patients post-intervention. This means that our analysis of barriers relating to access of women experiencing DVA is based on the narrative of HCPs expressing their views about those barriers. Another limitation is the small number of PIM respondents, precluding statistical analysis of changes in scores. In both the PIM responses and the interviews of HCPs, given that the interviewers were associated with the intervention, it is likely that social desirability bias was in play, exaggerating the acceptability of the intervention. Finally, our reliance on clinic registries for identification and referral data was a limitation, as consistent recording was hampered by the pressures of the COVID-19 pandemic and a prolonged nurses strike. This means that there was an under-recording of identification during the intervention.

## Conclusion

Primary health care clinics, with their recurrent contact with the population, are a suitable place for early intervention for victims and survivors of domestic violence in the oPT and globally. HCP in those clinics require training, clear referral pathways, increased safety, and leadership support and follow up to undertake this sensitive work. The HERA intervention needs further development, further clarifying roles in the care pathway, strengthening management/leadership support for staff in GBV focal points and case managers, and better integration of those staff into the training of HCP. Further development will need to be informed by the views of patients and all clinic team members. Finally, there is a need to expand DV training and referral pathways to hospitals, the setting for the majority of acute DV presentations.

## Electronic supplementary material

Below is the link to the electronic supplementary material.

Supplementary Material 1

Supplementary Material 2

Supplementary Material 3

Supplementary Material 4

## Data Availability

An aggregate summary of the data generated during this study is included in this published article. Individual data transcripts cannot be shared publicly due to confidentiality.

## Footnotes

1 * As of the 30th May 2024, after seven months of war, only 40% of primary care clinics were functional in Gaza [28].

## References

[1] World Health Organization. Global and Regional Estimates of Violence against Women. In: Prevalence and Health Effects of Intimate Partner Violence and Non-Partner Sexual Violence 2018 [Available from: https://www.who.int/publications/i/item/9789241564625

[2] Shaheen A, Ashkar S, Alkaiyat A, Bacchus L, Colombini M, Feder G, Evans M. Barriers to women’s disclosure of domestic violence in health services in Palestine: qualitative interview-based study. BMC Public Health. 2020;20(1):1795. 33243196 10.1186/s12889-020-09907-8 PMC7691108

[3] Clark CJ, Everson-Rose SA, Suglia SF, Btoush R, Alonso A, Haj-Yahia MM. Association between exposure to political violence and intimate-partner violence in the occupied Palestinian territory: a cross-sectional study. Lancet. 2010;375(9711):310–6. 20109958 10.1016/S0140-6736(09)61827-4

[4] Oxfam Italy. Baseline Study-Occupied Palestinian Territories, Naseej: Connecting Voices and Action to End Violence Against Women and Girls in the MENA Region. 2018.

[5] Garcia-Moreno C, Hegarty K, d’Oliveira AF, Koziol-McLain J, Colombini M, Feder G. The health-systems response to violence against women. Lancet. 2015;385(9977):1567–79. 25467583 10.1016/S0140-6736(14)61837-7

[6] World Health Organization. Sixty-ninth World Health Assembly, Geneva, 23–28 May 2016: resolutions and decisions, annexes 2016 [Available from: https://apps.who.int/iris/handle/10665/259134

[7] AWRAD (Arab World for Research and Development). Comprehensive Analysis for Gender Based Violence and the Status of the National Referral System in the West Bank. 2016.

[8] Airifai A. The health system and the enforcement of National Referral System for women survivors of violence: achievements, opportunities and process. Report by the Palestinian Ministry of Health and UNFPA. 2017.

[9] Colombini M, Alkaiyat A, Shaheen A, Garcia Moreno C, Feder G, Bacchus L. Exploring health system readiness for adopting interventions to address intimate partner violence: a case study from the occupied Palestinian territory. Health Policy Plan. 2020;35(3):245–56. 31828339 10.1093/heapol/czz151 PMC7152725

[10] Feder G, Davies RA, Baird K, Dunne D, Eldridge S, Griffiths C, et al. Identification and referral to Improve Safety (IRIS) of women experiencing domestic violence with a primary care training and support programme: a cluster randomised controlled trial. Lancet. 2011;378(9805):1788–95. 22000683 10.1016/S0140-6736(11)61179-3

[11] Sohal AH, Feder G, Boomla K, Dowrick A, Hooper R, Howell A, et al. Improving the healthcare response to domestic violence and abuse in UK primary care: interrupted time series evaluation of a system-level training and support programme. BMC Med. 2020;18(1):48. 32131828 10.1186/s12916-020-1506-3 PMC7057596

[12] Bacchus LJ, Alkaiyat A, Shaheen A, Alkhayyat AS, Owda H, Halaseh R, et al. Adaptive work in the primary health care response to domestic violence in occupied Palestinian territory: a qualitative evaluation using extended normalisation process theory. BMC Fam Pract. 2021;22(1):3. 33388033 10.1186/s12875-020-01338-z PMC7777212

[13] Bacchus LJ, d’Oliveira A, Pereira S, Schraiber LB, Aguiar JM, Graglia CGV, et al. An evidence-based primary health care intervention to address domestic violence against women in Brazil: a mixed method evaluation. BMC Prim Care. 2023;24(1):198. 37749549 10.1186/s12875-023-02150-1 PMC10519067

[14] University of Bristol. HERA - Healthcare Responding to Violence and Abuse 2022 [Available from: https://www.bristol.ac.uk/primaryhealthcare/researchthemes/hera/

[15] World Health Organization. Health care for women subjected to intimate partner violence or sexual violence: A clinical handbook 2013 [Available from: https://apps.who.int/iris/bitstream/handle/10665/136101/WHO_RHR_14.26_eng.pdf

[16] McKim CA. The value of mixed methods research: a mixed methods study. J Mixed Methods Res. 2017;11(2):202–22.

[17] Williamson E, Jones SK, Ferrari G, Debbonaire T, Feder G, Hester M. Health professionals responding to men for safety (HERMES): feasibility of a general practice training intervention to improve the response to male patients who have experienced or perpetrated domestic violence and abuse. Prim Health Care Res Dev. 2015;16(3):281–8. 25248144 10.1017/S1463423614000358

[18] Ryan K, Gannon-Slater N, Culbertson MJ. Improving Survey methods with cognitive interviews in small- and medium-scale evaluations. Am J Evaluation. 2012;33(3):414–30.

[19] Fetters MD, Curry LA, Creswell JW. Achieving integration in mixed methods designs-principles and practices. Health Serv Res. 2013;48(6 Pt 2):2134–56. 24279835 10.1111/1475-6773.12117 PMC4097839

[20] Green J, Thorogood N. Qualitative methods for health research. 2nd ed. Los Angeles: SAGE; 2009. xv, 304.

[21] Braun V, Clarke V. Using thematic analysis in psychology. Qual Res Psychol. 2006;3(2):77–101. 10.1191/1478088706qp063oa.

[22] De Silva MJ, Breuer E, Lee L, Asher L, Chowdhary N, Lund C, Patel V. Theory of change: a theory-driven approach to enhance the Medical Research Council’s framework for complex interventions. Trials. 2014;15:267. 24996765 10.1186/1745-6215-15-267 PMC4227087

[23] Hegarty K, McKibbin G, Hameed M, Koziol-McLain J, Feder G, Tarzia L, Hooker L. Health practitioners’ readiness to address domestic violence and abuse: a qualitative meta-synthesis. PLoS ONE. 2020;15(6):e0234067. 32544160 10.1371/journal.pone.0234067 PMC7297351

[24] Beynon CE, Gutmanis IA, Tutty LM, Wathen CN, MacMillan HL. Why physicians and nurses ask (or don’t) about partner violence: a qualitative analysis. BMC Public Health. 2012;12:473. 22721371 10.1186/1471-2458-12-473 PMC3444396

[25] Kalra N, Hooker L, Reisenhofer S, Di Tanna GL, Garcia-Moreno C. Training healthcare providers to respond to intimate partner violence against women. Cochrane Database Syst Rev. 2021;5(5):CD012423. 34057734 10.1002/14651858.CD012423.pub2 PMC8166264

[26] Colombini M, Mayhew SH, Garcia-Moreno C, d’Oliveira AF, Feder G, Bacchus LJ. Improving health system readiness to address violence against women and girls: a conceptual framework. BMC Health Serv Res. 2022;22(1):1429. 36443825 10.1186/s12913-022-08826-1 PMC9703415

[27] Szilassy E, Barbosa EC, Dixon S, Feder G, Griffiths C, Johnson M, et al. PRimary care rEsponse to domestic violence and abuse in the COvid-19 panDEmic (PRECODE): protocol of a rapid mixed-methods study in the UK. BMC Fam Pract. 2021;22(1):91. 33980165 10.1186/s12875-021-01447-3 PMC8115859

[28] World Health Organization. Emergency Situation Update (Issue 32) https://www.emro.who.int/images/stories/Sitrep_-_issue_32c.pdf?ua=1

