# Supplementary material for: Evaluation of a domestic violence training and support intervention in Palestinian primary care clinics in the west bank: a mixed method study
Source: BMC Prim Care. 2025 Apr 4;26:97. doi: 10.1186/s12875-025-02751-y (PMC11969874; doi:10.1186/s12875-025-02751-y)
Supplement: Supplementary file 1 — Supplementary Material 1 [file 12875_2025_2751_MOESM1_ESM.docx]

**Appendix**

*Table s1: How ready respondents felt to perform the following tasks when dealing with female patients who are experiencing domestic violence – before and after HERA.*

|  | **Pre PIM** | | | | | | |  | **Post PIM** | | | | | | |  | |
| --- | --- | --- | --- | --- | --- | --- | --- | --- | --- | --- | --- | --- | --- | --- | --- | --- | --- |
|  | **0**  **Not ready at all** | **1** | **2** | **3** | **4 Completely ready** | **N/A** | **Missing** | **Total** | **0**  **Not ready at all** | **1** | **2** | **3** | **4 Completely ready** | **N/A** | **Missing** | **Total** |  |
|  | **n** | **n** | **n** | **n** | **n** | **n** | **n** | **N** | **n** | **n** | **n** | **n** | **N** | **n** | **n** | **N** |  |
| Ask about domestic violence |  | 3 | 3 | 5 | 12 |  |  | 23 |  |  | 1 | 5 | 16 |  |  | 22 |  |
| Respond to disclosures of domestic violence |  | 2 | 2 | 6 | 13 |  |  | 23 |  |  |  | 5 | 17 |  |  | 22 |  |
| Identify signs and symptoms associated with domestic violence |  | 2 | 2 | 5 | 14 |  |  | 23 |  |  | 2 | 4 | 16 |  |  | 22 |  |
| Make referrals |  | 5 | 2 | 6 | 10 |  |  | 23 |  | 1 | 1 | 7 | 12 |  |  | 22 |  |
| Document disclosures of domestic violence |  | 4 | 1 | 3 | 15 |  |  | 23 |  | 1 | 3 | 4 | 14 |  |  | 22 |  |
| Provide ongoing support |  | 2 | 4 | 4 | 13 |  |  | 23 |  |  | 1 | 3 | 18 |  |  | 22 |  |

*Table s2: Pre, Post PIM medians and median difference*

| **Question** | **Medians of Pre PIM** | **Median of post PIM** | **Median difference** | **Interquartile range** |
| --- | --- | --- | --- | --- |
| This section asks how ready you feel now (after attending the HERA training) to perform the following tasks when dealing with female patients who are experiencing domestic violence. ‘0’ indicates feeling “Not ready at all” and 4 indicates feeling “Completely ready”. |  |  |  |  |
| 1. Ask about domestic violence | 4 | 4 | 0 | 0 |
| 1. Respond to disclosures of domestic violence | 4 | 4 | 0 | 0 |
| 1. Identify signs and symptoms associated with domestic violence | 4 | 4 | 0 | 0 |
| 1. Make referrals | 3 | 4 | 1 | 1 |
| 1. Document disclosures of domestic violence | 4 | 4 | 0 | 0 |
| 1. Provide ongoing support | 4 | 4 | 0 | 0 |
| 1. Discuss concerns about children living in a home where there is domestic violence | 4 | 4 | 0 | 0 |
| Q2. Do you feel afraid of dealing with a domestic violence case? 1) I feel very afraid  2) I feel moderately afraid  3) I do not feel afraid  4) I am not sure | 3 | 3 | 0 | 0 |
| Q3. To what extent do you feel protected by your organization/institution when dealing with a domestic violence case? 1) I feel very protected  2) I feel moderately protected  3) I do not feel protected  4) I am not sure | 3 | 3 | 0 | 0 |
| Q4. To what extent can you talk to women patients about domestic violence in a private and confidential space? (i.e. the conversation cannot be overheard and you are both alone)?  1) It is always possible to talk in a private and confidential space  2) In most cases it is possible to talk in a private and confidential space  3) It is rarely possible to talk in a private and confidential space  4) It is never possible to talk in a private and confidential space | 3 | 2 | -1 | -1 |
| Q5. To what extent can patient disclosures of domestic violence be kept confidential within the workplace (i.e. not discussed with people in the local community) 1) It is always possible to keep disclosures confidential  2) In most cases disclosures can be kept confidential  3) It is rarely possible to keep disclosures confidential  4) It is never possible to keep disclosures confidential | 1 | 1 | 0 | 0 |
| How many disclosures of domestic violence against women have been made to you in the last 6 months: Women who disclose that they are currently living with violence | 3.5 | 4 | 0.5 | 0.5 |
| Women who disclose that they have lived with violence in the past, but not currently: | 0 | 1 | 1 | 1 |
| Q7. How many disclosures of the following types of current CHILDHOOD ABUSE have been made to you in the X months: Childhood sexual abuse | 0 | 0 | 0 | 0 |
| Childhood physical abuse | 0 | 0 | 0 | 0 |
| Q8. In relation to CHILDREN who are living in a home with domestic violence, how many cases would you estimate you identified in the last X months? | 0 | 0 | 0 | 0 |
| Q10. Do you have any information on domestic violence and support services (e.g., leaflets, posters on walls, cards with contact numbers of services) available for women attending your clinic? 1) Yes, well displayed and accessible to patients  2) Yes, but not well displayed or accessible to patients 3) No  4) Not sure | 3 | 3 | 0 | 0 |

*Table s3: Pre and Post PIM results on “Do you feel afraid of dealing with a domestic violence case?”*

|  | **Pre PIM** | | **Post PIM** | |
| --- | --- | --- | --- | --- |
|  | **N** | **%** | **n** | **%** |
| I feel very afraid | 2 | 8.7% | 2 | 9% |
| I feel moderately afraid | 9 | 39% | 6 | 27% |
| I do not feel afraid | 10 | 43.4% | 14 | 63% |
| I am not sure | 2 | 8.7% |  |  |
| Missing/ ignored |  |  |  |  |
| **Total** | **23** | **100,0** | **22** | **100,0** |

*Table s4: To what extent do you feel protected by your organization/institution when dealing with a domestic violence case?*

|  | **Pre PIM** | | **Post PIM** | |
| --- | --- | --- | --- | --- |
|  | **N** | **%** | **n** | **%** |
| I feel very protected | 0 |  |  |  |
| I feel moderately protected | 5 | 21.7% | 4 | 18% |
| I do not feel protected | 12 | 52.1% | 16 | 72% |
| I am not sure | 6 | 26% | 2 | 9% |
| Missing/ ignored |  |  |  |  |
| **Total** | **23** | **100,0** | **22** | **100,0** |

*Table s5: To what extent can you talk to women patients about domestic violence in a private and confidential space? (i.e. the conversation cannot be overheard and you are both alone)?*

|  | **Pre PIM** | | **Post PIM** | |
| --- | --- | --- | --- | --- |
|  | **n** | **%** | **n** | **%** |
| It is *always possible* to talk in a private and confidential space | 4 | 17.4% | 7 | 31.*% |
| *In most cases* it is possible to talk in a private and confidential space | 6 | 26.1% | 5 | 22.7% |
| It is *rarely possible* to talk in a private and confidential space | 8 | 34.7% | 8 | 36% |
| It is *never possible* to talk in a private and confidential space | 5 | 21.7% | 1 | 4.5% |
| Missing/ ignored |  |  |  |  |
| **Total** | **23** | **100,0** | **22** | **100,0** |

*Table s6: To what extent can patient disclosures of domestic violence be kept confidential within the workplace (i.e. not discussed with people in the local community)*

|  | **Pre PIM** | | **Post PIM** | |
| --- | --- | --- | --- | --- |
|  | **n** | **%** | **n** | **%** |
| It is *always possible* to keep disclosures confidential | 20 | 86.9% | 15 | 68% |
| In *most cases* disclosures can be kept confidential | 2 | 8.7% | 7 | 31.8% |
| It is *rarely possible* to keep disclosures confidential | 1 | 4.3% |  |  |
| It is *never possible* to keep disclosures confidential |  |  |  |  |
| Missing/ ignored |  |  |  |  |
| **Total** | **23** | **100,0** | **22** | **100,0** |

*Table s7: Do you know of support services to which you can refer female patients who are experiencing domestic violence?*

|  | **Pre PIM** | | **Post PIM** | |
| --- | --- | --- | --- | --- |
|  | **n** | **%** | **n** | **%** |
| Yes | 15 | 65.2% | 21 | 95.4% |
| No | 7 | 30.4% | 1 | 4.5% |
| Not sure | 1 | 4.3% |  |  |
| Missing/ ignored |  |  |  |  |
| **Total** | **23** | **100,0** | **22** | **100,0** |


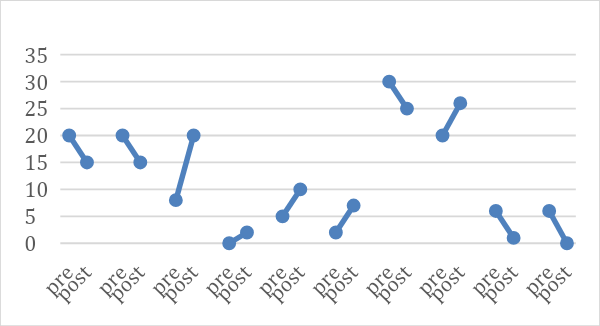


*Figure s1: Number of women who disclose that they are currently living with violence in matched pre and post PIM results*


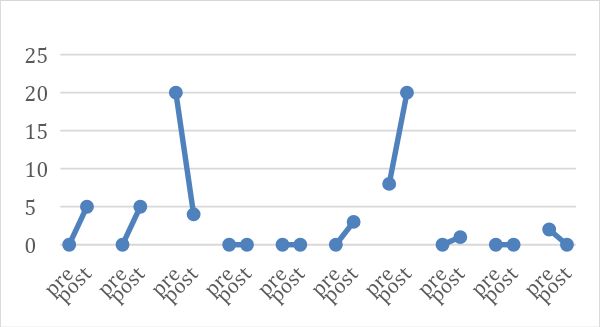


*Figure s2: Number of women who disclosed that they have lived with violence in the past, but not currently in matched pre and post PIM results*


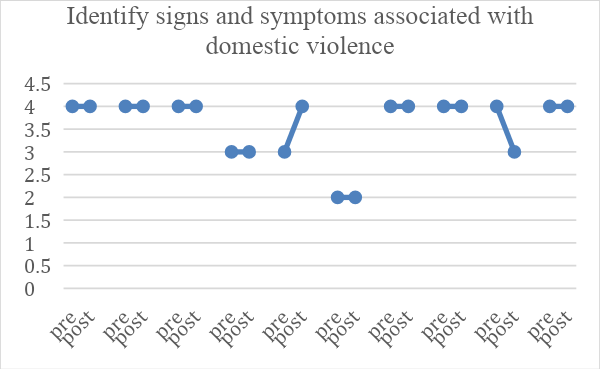


*Figure s3: Readiness to identify signs and symptoms associated with DV in matched pre and post PIM results - ‘0’ indicates feeling “Not ready at all” and 4 indicates feeling “Completely ready”.*


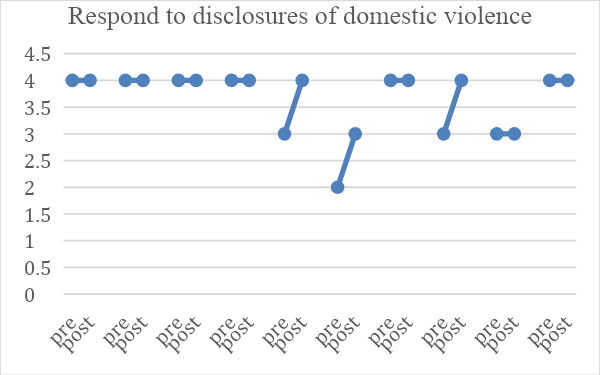


*Figure s4: Readiness to respond to disclosure of DV in matched pre and post PIM results - ‘0’ indicates feeling “Not ready at all” and 4 indicates feeling “Completely ready”.*


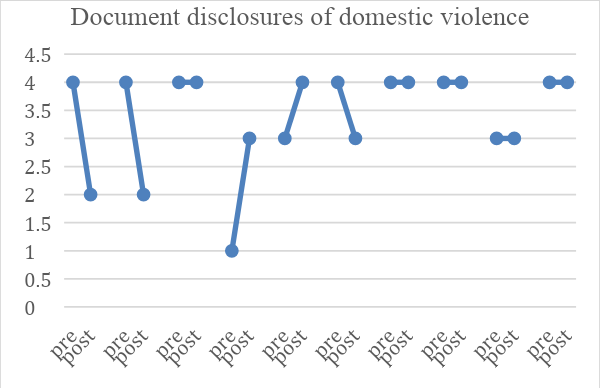


*Figure s5: Readiness to document disclosure of DV in matched pre and post PIM results -‘0’ indicates feeling “Not ready at all” and 4 indicates feeling “Completely ready”.*


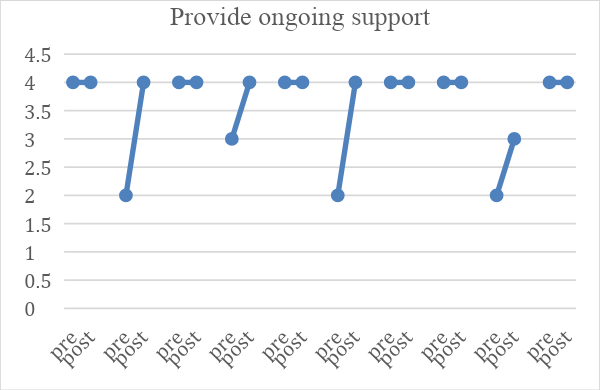


*Figure s6: Readiness to provide ongoing support in matched pre and post PIM results - ‘0’ indicates feeling “Not ready at all” and 4 indicates feeling “Completely ready”.*


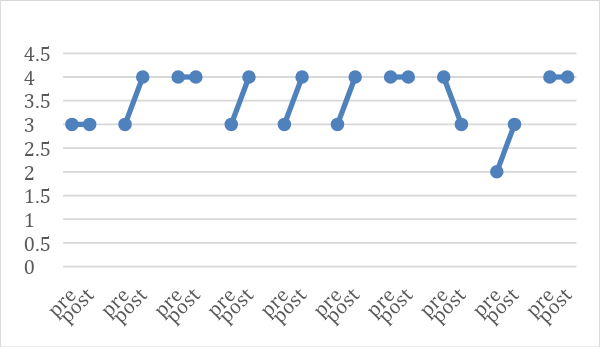


*Figure s7: Readiness to discuss concerns about children living in a home where there is domestic violence in matched pre and post PIM results - ‘0’ indicates feeling “Not ready at all” and 4 indicates feeling “Completely ready”.*


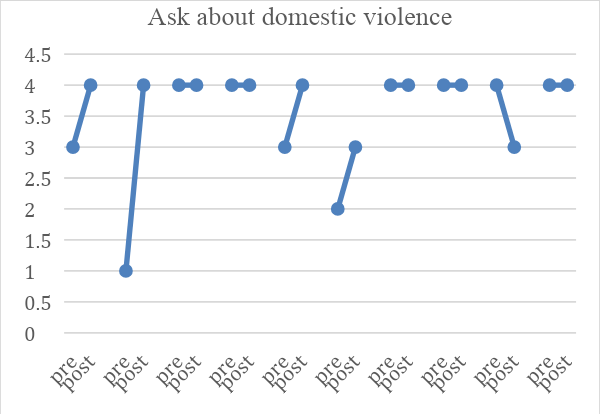


*Figure s8: Readiness to Ask about DV when dealing with women experiencing DV in matched pre and post PIM results*

*‘0’ indicates feeling “Not ready at all” and 4 indicates feeling “Completely ready”.*


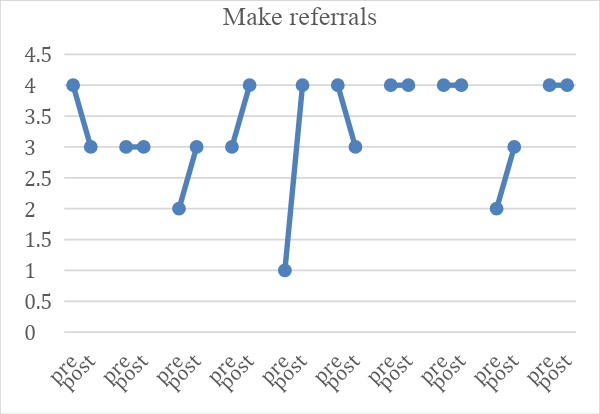


*Figure s9: Readiness to Make referrals in matched pre and post PIM results*

*‘0’ indicates feeling “Not ready at all” and 4 indicates feeling “Completely ready”.*


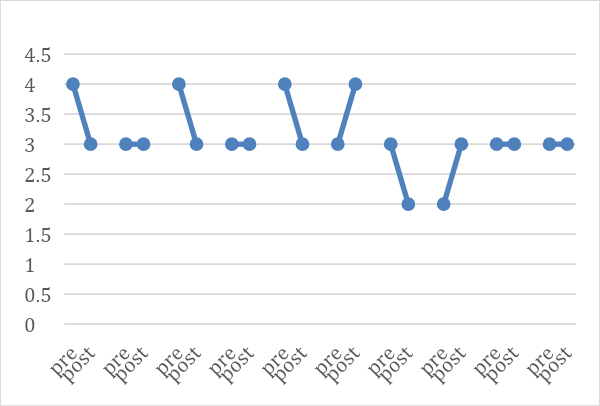


*Figure s10: To what extent do HCP feel protected by their organization/institution when dealing with a DV case? 1) very protected 2) moderately protected 3) do not feel protected 4) not sure*


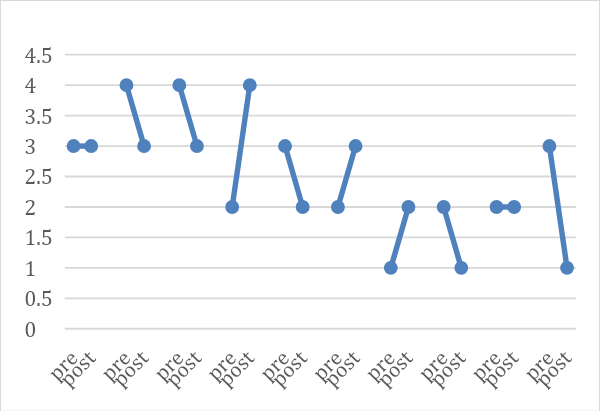


*Figure s11: The extent can they talk to women patients about DV in a private and confidential space.*

*1) always possible 2) In most cases 3) rarely possible 4) never possible*
