# Supplementary material for: Evaluation of a domestic violence training and support intervention in Palestinian primary care clinics in the west bank: a mixed method study
Source: BMC Prim Care. 2025 Apr 4;26:97. doi: 10.1186/s12875-025-02751-y (PMC11969874; doi:10.1186/s12875-025-02751-y)
Supplement: Supplementary file 3 — Supplementary Material 3 [file 12875_2025_2751_MOESM3_ESM.docx]

**PROVIDER INTERVENTION MEASURE (pre-PIM)**

**Thank you for agreeing to complete this survey.**

Your participation in this **brief survey** will help us to **evaluate and improve** the

**HERA (HEalthcare Responding to violence and Abuse) training intervention**.

We are inviting you to complete this survey before the first HERA training session and again X months later.

We are assessing the effects of the training - not you!

We are interested in the average learning and behavioural impact of the training sessions; we are not concerned with individual answers and individual results will be strictly confidential.

Any information you give us will be treated as confidential. We will not pass on any identifiable information in relation to your responses to your clinic, the trainers or anyone else. We will store information collected in the study in locked filing cabinets and on password protected databases at [NAME OF UNIVERSITY]. You can ask for your data to be removed from the study up to one month after completing the survey.

Please fill in your answers based on how confident and prepared you feel today.

Please answer as honestly as you can.

**SECTION 1: GENERAL DETAILS**

This section asks for general details about you. **All information will be treated in confidence and anonymised.**

**Please insert your unique identifier code**

Your unique identifier code will be the initials of your first and last name and the day of your birth (e.g. EW2802). This will allow us to match your second survey (administered X months after training) to explore changes.

**Please enter the date you are completing this survey (day, month and year)**

**Q1 What is your age?**

24 or under ☐

25-29 ☐

30-34 ☐

35-39 ☐

40-44 ☐

50-54 ☐

45-49 ☐

55-59 ☐

60-64 ☐

65-69 ☐

70 or over ☐

**Q2 Sex**

Female ☐

Male ☐

Other (please specify)

**Q3 What is your job title?**

General MD ☐

Specialist MD ☐

If specialist MD, please specify your specialty …………………………………………………………..

Staff Nurse ☐

Practical Nurse ☐

Midwife ☐

Administrative ☐

Other ☐

Reception staff ☐

Clinic/Hospital Director ☐

Health care manager ☐

If ‘other’ please specify …………………………………………………………………………………………

**Q4 How many years have you worked in the health care sector?**

**Q5 For how many years have you worked in a Primary Health Care setting/Outreach Clinic/Sexual and Reproductive Health Care setting?**

**Q6 Average number of patients you come into contact with (i.e. face-to-face contact) per day?**

**SECTION 2: BACKGROUND**

This section asks about the training you may have attended addressing domestic violence. Although some of the questions are similar it is important for us to know what kind of training you may, or may not, have received.

Throughout this survey, the term ***domestic violence*** is used in relation to adult women victims (i.e. aged 18 and over).This refers to any act or failure to act of a household member against another member in the household for the purpose of causing physical, sexual or psychological abuse, or the threat of physical, sexual or psychological abuse, or to generate fear. It also includes the deprivation of basic rights such as shelter, food, drink, clothing, education, freedom of movement and loss of self-determination and self-security. It includes violence from a spouse or other family member (Palestine National Authority, Palestinian Central Bureau of Statistics, 2011).

**Q7 Have you had any training on domestic violence? *(Please tick all that apply)***

***If you have never received training on domestic violence please go straight to question Q8***

| **Type of training** | **Yes** |
| --- | --- |
| Watched a video |  |
| Completed a web-based programme |  |
| Attended a lecture or talk |  |
| Medical/nursing/other school-classroom training |  |
| Medical/nursing/other school-clinical setting |  |
| Registrar/other post-graduate training |  |
| Other in-depth training (more than 4hours) |  |
| Other (please specify name of the institution who conducted the training) |  |

**Q7a Did any of this previous training include information about any of the following?**

**(For the purposes of this survey, a child or young person is anyone under the age of 18)**

|  | Yes | No | Not sure |
| --- | --- | --- | --- |
| Specific role and actions of primary health care professionals? |  |  |  |
| Children living in a home where there is domestic violence |  |  |  |
| Children who are directly abused by someone in the home |  |  |  |

**Q8 Please tick the number that best indicates *how ready you feel* to perform the following tasks when dealing with female patients who are experiencing domestic violence. ‘0’ indicates feeling “Not ready at all” and 4 indicates feeling “Completely ready”.**

**Please respond to as many items as possible. If an item does not apply to you (example, you are a receptionist or administrator) please tick the ‘Not Applicable’ box**

|  | **0**  **Not ready at all** | **1** | **2** | **3** | **4**  **Completely ready** | Not  Applicable |
| --- | --- | --- | --- | --- | --- | --- |
| Ask about domestic violence |  |  |  |  |  |  |
| Respond to disclosures of domestic violence |  |  |  |  |  |  |
| Identify signs and symptoms associated with domestic violence |  |  |  |  |  |  |
| Make referrals |  |  |  |  |  |  |
| Document disclosures of domestic violence |  |  |  |  |  |  |
| Provide ongoing support |  |  |  |  |  |  |
| Discuss concerns about children living in a home where there is domestic violence |  |  |  |  |  |  |

**Q9a. Do you feel afraid of dealing with a domestic violence case?**

I feel very afraid                               ☐

I feel moderately afraid                 ☐

I do not feel afraid                           ☐

I am not sure                                     ☐

**Q9b. To what extent do you feel protected by your organization/institution when dealing with a domestic violence case?**

I feel very protected                     ☐

I feel moderately protected        ☐

I do not feel protected                 ☐

I am not sure                                 ☐

**Q10 To what extent can you talk to women patients about domestic violence in a private and confidential space? (i.e. the conversation cannot be overheard and you are both alone)?**

It is *always possible* to talk in a private and confidential space ☐

*In most cases* it is possible to talk in a private and confidential space ☐

It is *rarely possible* to talk in a private and confidential space ☐

It is *never possible* to talk in a private and confidential space ☐

**Q11 To what extent can patient disclosures of domestic violence be kept confidential within the workplace (i.e. not discussed with people in the local community)**

It is *always possible* to keep disclosures confidential ☐

In *most cases* disclosures can be kept confidential ☐

It is *rarely possible* to keep disclosures confidential ☐

It is *never possible* to keep disclosures confidential ☐

**SECTION 3: CURRENT PRACTICE**

This section deals with what you currently do in your workplace. Your responses will be treated anonymously so please be as honest as possible in your answers.

**Q12. How many disclosures of domestic violence against women have been made to you in the last X months:**

|  | Please write the number |
| --- | --- |
| Women who disclose that they are currently living with violence |  |
| Women who disclose that they have lived with violence in the past, but not currently |  |

**Q13 How many disclosures of the following types of current CHILDHOOD ABUSE have been made to you in the X months:**

|  | Please write the number |
| --- | --- |
| Childhood sexual abuse |  |
| Childhood physical abuse |  |

**Q14 In relation to CHILDREN who are living in a home with domestic violence, how many cases would you estimate you identified in the last X months?**

|  |
| --- |

**Q15 How often, in the past 6 months, have you asked a female patient about domestic violence when they have presented with the following conditions:**

|  | Have not seen this presentation in the last 6 months | Never | Rarely | Sometimes | Nearly always | Always |
| --- | --- | --- | --- | --- | --- | --- |
| Physical injuries |  |  |  |  |  |  |
| Chronic pelvic pain |  |  |  |  |  |  |
| Dyspareunia (painful sex) |  |  |  |  |  |  |
| Irritable bowel syndrome |  |  |  |  |  |  |
| Headaches |  |  |  |  |  |  |
| Depression/anxiety |  |  |  |  |  |  |
| Hypertension |  |  |  |  |  |  |
| Eating disorders |  |  |  |  |  |  |
| Sleep disruption |  |  |  |  |  |  |
| General stress |  |  |  |  |  |  |
| STIs |  |  |  |  |  |  |
| Frequent vague complaints |  |  |  |  |  |  |
| Patient looks upset, sad, has unkempt appearance or other behavioural cues |  |  |  |  |  |  |
| Patient has a disability or impairment |  |  |  |  |  |  |

**Q16 Do you have any information on domestic violence and support services (e.g. leaflets, posters on walls, cards with contact numbers of services) *available for women* attending your clinic?**

Yes, well displayed and accessible to patients ☐

Yes, but not well displayed or accessible to patients ☐

No ☐

Not sure ☐

**Q17 Do you have any information on domestic violence specifically *for health care* *providers* to use during consultations with women? (e.g. to guide discussions about domestic violence, to know about referral services, safety planning etc…)**

Yes ☐

No ☐

Not sure ☐

**Q17a Do you know of support services to which you can refer female patients who are experiencing domestic violence?**

Yes ☐ Go to question Q17b

No ☐

Not sure ☐

**Q17b If yes, what services do you know about? Please tick all that apply.**

**Each country can have a list that is specific to their context. You can include those within the health system/clinic and those that are external. Some examples….**

**Within the health care system**

Psychologist ☐

Social worker ☐

NPV ☐

GBV Focal Point ☐

OMCs ☐

**Outside the health care system**

Shelters ☐

xxxx

If you have any additional comments, please use the text box below or you can email the [P](mailto:principal)rincipal Investigator of the study.

**Name of PI and affiliation, email and telephone number here**

**Thank you for your assistance. We will be contacting you again in X months to complete a**

**follow-up survey**
