# Supplementary material for: Evaluation of a domestic violence training and support intervention in Palestinian primary care clinics in the west bank: a mixed method study
Source: BMC Prim Care. 2025 Apr 4;26:97. doi: 10.1186/s12875-025-02751-y (PMC11969874; doi:10.1186/s12875-025-02751-y)
Supplement: Supplementary file 4 — Supplementary Material 4 [file 12875_2025_2751_MOESM4_ESM.docx]

**HERA 2 Topic Guide for Primary Healthcare Providers Post HERA Intervention**

**Thank you for agreeing to participate in a follow-up interview. We are interested in hearing about your experiences since attending the HERA (Healthcare Responding to Violence and Abuse) training intervention. This will help us to improve the training and better support primary health care providers in the management of domestic violence. Please be assured that your answers are completely confidential and we will not pass your responses on to the clinic, the trainers or anyone else. Please answer as honestly as you can.**

**First, before we start the interview, I would like to ask a few questions that help to describe you.**

Respondent unique ID number [note: it’s useful to use a system to match providers who completed a qualitative interview in phase 1 and phase 2 (e.g. HP01_P1 and HP01_P2) and/or pre and post PIM e.g., HP01_Pre, HP01_Post]:

Age:

Sex:

Ethnic group:

Position/Job title:

Name of Facility:

How many hours/days do you normally work at the facility:

Name of interviewer:

Date of interview:

**Views on HERA training and training team**

1. What are your views of the HERA training?

**Probes:**

- - In what ways has the training prepared you for for dealing with DV cases. Ask for examples (e.g. comfort in asking questions, in offering support and dealing with women’s requests, dealing with other family members, documentation)
  - What could be improved? Ask for examples
  - What did you want more of? Ask for examples of topics
  - What are your views on the training model (i.e. web-based and face-to-face)? - What worked well/what didn’t work so well, and why? Ask for examples (e.g. internet connection, difficulty understanding topics or doing group work, maintaining concentration, time, any others)
  - In your clinic, how easy or difficult has it been to develop a team response to domestic violence using mostly web-based training?
  - What support have staff been given to participate in the initial and follow-up training sessions (e.g. dedicated time during working hours, data allowance, laptops, space)?
  - To what extent has Covid-19 affected your ability to participate in the training sessions? Can you tell me more?

1. What are your views and experiences of the training team and the way the initial and follow-up sessions were delivered? For example....

**Probes:**

- Their ability to facilitate web-based training versus face to face?
- Their knowledge and expertise
- Their ability to deal with questions and concerns
- The balance of information giving versus practical skills
- The ongoing support and training activities they offered to providers

**Impact of HERA training on dealing with domestic violence cases**

1. Since attending the HERA training, have you dealt with a case of domestic violence and can you tell me about it?

*Note: if they haven’t encountered a case personally, ask if they know about any cases identified in the clinic and try to adapt the questions as far as possible.*

**Probes:**

- Can you tell me how the disclosure of domestic violence occurred? What you said/what she said?
  - Explore their comfort in discussing domestic violence
- What kinds of help did the woman ask for - for herself, for her husband, for her children? (e.g. listening and talking, practical help, keeping family together, talk to the partner/husband, help for alcohol/drug issues, referrals to other services – ask what types)
- Did you feel that any of the help she asked for was beyond your role as a health care provider? Tell me more about that?
- How did you respond in this case? Were any other providers in the clinic involved in this case and if so, what did they do? (e.g. explore roles, referrals, documentation, follow-up)
- Since the HERA training, what have been the ongoing challenges of dealing with cases of domestic violence? (Covid-19, time, following up cases, fears about personal safety, women not wanting referrals, lack of confidence in skills, any others?)

1. Have you encountered any female patients who reported experiencing any partner-or family-related barriers (including physical or /and emotional violence) to access to or use of contraception?

**Probes:**

- - [If yes] What experiences can you share?
    1. Issues to explore: details about the type of barriers and violence (e.g. any physical, or emotional, including threats, violence from their male partners or others); explore their opinion about such barriers;
    2. What was your approach to discussing these?
  - **[If not encountered]** What do you think you could do?

1. How do you feel about your personal safety when dealing with domestic violence cases since attending the HERA training?

**Probes:**

- To what extent has HERA training helped to address providers’ fears in dealing with domestic violence cases?
- Can you share any examples in the clinic where provider safety has been compromised when dealing with a domestic violence case?
- What needs to be in place to help providers feel safer and more protected at work when dealing with DV cases? And who should be responsible for that?

**Views on the referral pathway and roles within it**

1. Since the HERA training, what is your understanding of the different roles of clinic staff and what they do, including your own role?

**Probes:**

- e.g. GBV focal point/Clinic Case Officer/NPV [please adapt the names of roles to country context)

1. How well has the referral pathway worked in reality?

**Probes:**

- Any challenges and with whom? (specify roles, any examples)
- To what extent has it helped with coordination and follow-up of cases?

**If they say they have not seen *any* DV cases since training ask:**

- How well do you think these roles *would work* in practice?

1. What support was available to you after the HERA training?

**Probes:**

- What kind of support were you given and by who? Give examples
- Have you found any changes in the support offered by clinic/managers/other colleagues? If so, give examples
- What kind of support do you think should be in place for providers and who should provide this?

**Covid-19 and lock down**

1. How has Covid-19 affected the the clinic’s routine work?

**Probes**

- How has it affected your own work? (ask for example of challenges)
- What strategies are being used/or should be used to overcome these challenges (if any challenges mentioned above)

1. How has Covid-19 and the lock down affected women who are experiencing domestic violence? (e.g. women’s ability to get to the clinic or come for follow-ups, getting to other services, women’s coping mechanisms, economic strain).
2. Since Covid-19, have you noticed an increase or decrease in the numbers of domestic violence cases at the clinic?
   - If yes, why do you think this is?

If you’ve not seen any domestic violence cases at the clinic, where do you think women are going to seek help?

1. Can you tell me about any experiences you’ve had of offering remote support to women experiencing domestic violence – for example through a telephone consultation? (Explore any difficulties they had, how they dealt with them, how does it compare to in-person support).

**Other contextual factors (other than Covid 19)**

**To country teams: for the synthesis package on implementation in different settings, we are interested in capturing wider contextual issues (beyond what happens in the clinic or health system) that might have a direct or indirect impact on HERA. Although it’s beyond the control of our intervention to address these issues, it is still important to capture them as they always interact with the intervention. Some examples are below, but you are best placed to identify these factors and develop some questions. For example, austerity measures implemented by governments or deprioritising gender issues (e.g. Brazil), political occupation decreasing security for women/providers (oPt).**

1. What other contextual factors (other than Covid) do you think may have impacted on the HERA intervention?
   - Terrorist attacks
   - Elections and change of government
   - Autsterity policies
   - Ongoing political occupation oPT

**Probes**

- Try to explore using your own probes whether and how all these wider contextual factors are impacting on each other and the HERA intervention.

**Violence against children**

1. Since attending the HERA training have you encountered any cases of children being directly abused by someone in the home, or children being exposed to domestic violence at home?

**Probes:**

- Explore the context in which they became aware of the case
- What actions did they and others in the clinic take (e.g. roles re: referral, follow-up)
- What difficulties, if any, did they encounter and how did they deal with it
- To what extent did HERA training prepare them to deal with such cases

**Those are all the questions I have for you. Is there anything you would like to add? Thank you, I appreciate the time you have given and feedback for the research study.**
